# Malaria treatment for prevention: a modelling study of the impact of routine case management on malaria prevalence and burden

**DOI:** 10.1186/s12879-024-09912-x

**Published:** 2024-11-08

**Authors:** Flavia Camponovo, Aurélie Jeandron, Laura A Skrip, Monica Golumbeanu, Clara Champagne, Tasmin L Symons, Mark Connell, Peter W Gething, Theodoor Visser, Arnaud Le Menach, Justin M Cohen, Emilie Pothin

**Affiliations:** 1 Swiss Tropical and Public Health Institute, Basel, Switzerland; 2 University of Basel, Basel, Switzerland; 3 University of Liberia School of Public Health, Monrovia, Liberia; 4 Telethon Kids Institute, Perth Children’s Hospital, Perth, Australia; 5 School of Population Health, Curtin University, Perth, Australia; 6 Clinton Health Access Initiative, Boston, USA

**Keywords:** P. Falciparum, Malaria, Treatment, Modelling, Transmission

## Abstract

**Background:**

Testing and treating symptomatic malaria cases is crucial for case management, but it may also prevent future illness by reducing mean infection duration. Measuring the impact of effective treatment on burden and transmission via field studies or routine surveillance systems is difficult and potentially unethical. This project uses mathematical modeling to explore how increasing treatment of symptomatic cases impacts malaria prevalence and incidence.

**Methods:**

Leveraging the OpenMalaria stochastic agent-based transmission model, we first simulated an array of transmission intensities with baseline effective treatment coverages of 28%, 44%, and 54% incorporated to reflect the 2023 coverage distribution across Africa, as estimated by the Malaria Atlas Project. We assessed the impact of increasing coverage to as high as 60%, the highest 2023 estimate on the continent. Subsequently, we performed simulations resembling the specific subnational endemicities of Kenya, Mozambique, and Benin, using the Malaria Atlas Project estimates of intervention coverages to reproduce historical subnational prevalence. We estimated the impact of increasing effective treatment coverage in these example settings in terms of prevalence reduction and clinical cases averted in children under 5 years old and the total population.

**Results:**

The most significant prevalence reduction – up to 50% – was observed in young children from lower transmission settings (prevalence below 0.2), alongside a 35% reduction in incidence, when increasing effective treatment from 28% to 60%. A nonlinear relationship between baseline transmission intensity and the impact of treatment was observed. Increasing effective treatment coverage to 60% reduced the risk in high-risk areas (prevalence in children under 5 years old > 0.3), affecting 39% of young children in Benin and 20% in Mozambique previously living in those areas. In Kenya where most of the population lives in areas with prevalence below 0.15, and case management is fairly high (53.9%), 0.39% of children were estimated to transition to lower-risk areas.

**Conclusions:**

Improving case management directly reduces the burden of illness, but these results suggest it also reduces transmission, especially for young children. With vector control interventions, enhancing case management can be an important tool for reducing transmission intensity over time.

**Supplementary Information:**

The online version contains supplementary material available at 10.1186/s12879-024-09912-x.

## Background

Malaria remains a leading cause of morbidity and mortality globally, and although several countries recently reached elimination [1] or are close to elimination [2], malaria endemicity remains a challenge across the globe. The African continent bears the greatest malaria burden with 93.6% of the world’s total cases in 2022, and with an estimated incidence of 222.6 per 1000 population at risk [3]. The highest burden remains in children under 5 years old, with 78.1% of malaria deaths in Africa occurring in that age group [3]. Many interventions have been designed and implemented to reduce malaria burden and transmission, including tools focusing on reducing the mosquito population, preventing the vector-host interactions, and preventing or treating human infections [3]. Routine malaria case management, consisting of early diagnosis and treatment of malaria, is an essential component of malaria control [4]. Three-day artemisinin-combination therapies (ACTs) are the recommended first-line treatment for uncomplicated malaria [4]. Even though the drug efficacies are high [5, 6], many are unable to access them due to health system gaps, and effectiveness may be reduced by lack of compliance or non-adherence [7].

The overall health system performance for malaria case management in a given place can be quantified with the metric of effective treatment coverage. Effective treatment coverage accounts for the various components within the cascade of care, including access to care from a formal or informal provider, the availability of and provider’s compliance to recommended treatment, the patient’s adherence to treatment regimen, and the drug’s cure rate, which depends on the drug’s efficacy and possible parasite resistance [7]. Across African countries, the latest estimates of effective treatment coverage range from 6 to 61%, with on average 60% of the population without effective access to care across the malaria endemic African regions [8], showing both the wide heterogeneity between countries and the potential for improvement in many countries.

*P. falciparum* malaria infections are defined by their chronic nature, with untreated infections capable of lasting several months [9]. As such, treating symptomatic malaria cases is expected to reduce disease incidence and mortality. Additionally, by clearing the infection shortly after symptom onset and thus drastically shortening the length of infection, it might have the added benefit of reducing the overall transmission potential in the population. Measuring the effect of treating symptomatic infections in a classical trial which includes an untreated control group is impossible for obvious ethical reasons. And because national malaria control programs in endemic countries rarely provide case management services in isolation, estimating the effect of case management on burden and transmission via clinical trial or routine surveillance systems is a challenge.

Mathematical modelling of disease transmission has become an essential tool to investigate complex disease dynamics [10, 11] not measurable in real life. Models are often used to investigate the impact of novel interventions or intervention scenarios on disease burden and transmission. In the context of *P. falciparum*, for example, modeling studies have estimated the public health impact of RTS, S/AS01 immunization strategies across Sub-Saharan Africa [12], the impact of mass drug administration on transmission in settings close to elimination [13], the role of case management in controlling imported infection [14], the importance of improved diagnostics in mass screen and treat strategies [15], or the effect of mass distribution of bednets on transmission [16]. In parallel to the development and use of malaria disease models, statistical methods to estimate essential parameters to calibrate simulations to country-like settings have been developed, and publicly available databases are continuously updated. These include global demographic estimates from WorldPop [17] and global malaria prevalence estimate as well as malaria intervention coverages from the Malaria Atlas Project [18].

To overcome the complexities in assessing routine case management impact on transmission in the field, we use mathematical modelling of malaria transmission to investigate the potential impact of increasing effective treatment coverage across a range of endemic settings.

## Methods

### OpenMalaria model

This study uses OpenMalaria (version 44), an agent-based model of malaria transmission, detailed in [19, 20]. Briefly, it combines a deterministic, periodically-forced difference equation model of the vector life-cycle [21] – where new mosquitos emerge from water bodies, actively search for blood meals, encounter and feed on different types of hosts, search for a resting place, and lay eggs before seeking for new hosts [22] – with an individual based model of *P. falciparum* parasite population in humans, updated in a 5-day time step [19]. Infection of humans depends on the age specific entomological inoculation rate (EIR), and accounts for a decreased success of inoculation with increased EIR due to innate and acquired immunity [23]. Asexual blood-stage infections were calibrated using historical data from patients infected with *P. falciparum* as treatment for neurosyphilis, which show large variation of infection lengths (median 205 days, minimum and maximum 37 and 405 days) [24] and are characterized by stochastic variation in infection duration and parasite densities, with a general decrease in parasitemia over time of infection, and inter-individual variation [25]. Acquired asexual immunity act on parasite densities and results from cumulative density of asexual parasites (since birth), the cumulative number of prior infections that the host experienced, and maternal immunity in babies [23, 26]. Infectiousness to a feeding mosquito depend on parasite densities 10, 15 and 20 days previously, allowing time for gametocytes to develop and circulate, and the probability that at least one male and one female gametocyte is taken up in the blood meal [27]. A pathogenesis model is used to estimate incidence of uncomplicated and severe cases of malaria, with the probability of acute morbidity dependent on the asexual density and a pyrogenic threshold, the latter increasing with the host’s past exposure as an implicit result of immune response. Severe malaria results from high parasite load [28, 29].The probability that an uncomplicated malaria case is successfully treated equals the effective access to treatment estimate. If a case is treated, asexual parasites are cleared in the next time step (5 days), with no additional prophylactic effect and without explicitly defining the type of drug nor its efficacy profile.

Simulation fits of key indicators can be found in the initial publications (collected in the supplement to the American Journal of Tropical Medicine and Hygiene, Volume 75: Issue 2 supplement). In particular, plots of calibrations with data across multiple African sites are shown for age-specific indicators for prevalence [23, 26, 29], incidence [23, 29], severe incidence and severe incidence-prevalence curves [28], and parasite density [26, 29]. Relationships between body surface area and mosquito biting rates, as well as incidence-EIR relationships, are found in [23], and the fits for parasitemia and maximum parasite densities in the first wave of parasitemia in malaria therapy patients are found in [23, 25]. A summary of the extensive datasets used for calibration can be found in [20].

### Effective access to treatment estimates

Effective access to treatment is defined as effectively clearing blood-stage malaria parasites upon receiving treatment. Estimates of effective access to treatment at admin-1 level (administrative boundaries of the first sub-national level) are taken from the Malaria Atlas Project (MAP), and are defined as a composite of modelled rates of care-seeking [30], proportional use of ACT versus non-ACT drug classes, and the effectiveness of each drug class [31], thus estimating the proportion of the population with access to curative treatment for uncomplicated malaria. Data and models are stratified by sector, reflecting differential rates in ACT uptake [31].

### Simulations in hypothetical settings

Using OpenMalaria, we first explored more generally how the changes in case management impact malaria burden across different transmission levels. For this purpose, we built a set of “hypothetical” malaria settings starting from the characteristics of the Alibori department in Benin in terms of demography, seasonality and vector composition [32]. The model was simulated for yearly EIR between 1 and 320 (with a 0.5 step), representing PfPR_2 − 10_ levels between [0.003–0.6], to investigate the impact of case management across the full range of transmission intensities. A seasonality pattern with a transmission peak in October was assumed. Three baseline effective treatment coverages were assumed, 28%, 44%, and 54%. These values represent the median and interquartile range of 2023 estimates across African admin-1 units in 2023 from MAP [8]. The model was run over 30 years. The simulation was initiated with no case management; then, a baseline level of case management was deployed after 10 years, for 13 years. A uniformly incremental increase in case management coverage was then implemented over 3 years to reach a new coverage, which then remained constant until the end of simulation time. The improved coverage was either set to the absolute target level of 60% – which represents the highest 2023 estimate from MAP across admin1 units, found in Uganda, and used as an operationally feasible best-case target – or it was set as an intermediate relative increase of either 50% or 75% from baseline coverage to the target level. No other interventions were implemented. Each scenario was simulated 10 times in a population of 10’000.

### Simulations resembling admin-1 regions of Benin, Kenya, and Mozambique

Next, the model simulations were updated to reflect country-specific data on case management and other malaria interventions. Specifically, the model was parameterized for the contexts of Benin, Kenya, and Mozambique, and run to capture sub-national variation. Simulations were performed at the admin-1 level - which represents administrative boundaries of the first sub-national level - corresponding to 12 regions in Benin, 48 in Kenya, and 11 and Mozambique. Precisely, for each administrative region within a country, yearly regional estimates between 2000 and 2022 of bednet coverage, indoor residual spraying (IRS) coverage, and effective treatment coverage were implemented in the model. These estimates were computed by the MAP [8]. In the model, bednets affect parameters in the model of mosquito feeding cycle by decreasing the availability of hosts to mosquitos, decreasing the probability that a mosquito bites the host upon encounter, and decreasing the probability that the mosquito finds a resting place after feeding [22]. The effect of IRS is translated in the model by decreasing the availability of hosts to mosquitos, and decreasing the probability that a mosquito survives in its resting place after feeding [22]. Pyrethroid insecticide treated bednets were parameterized previously and assume a high initial efficacy with mean insecticide concentration of 55.5mg.m^− 2^ (SD 14), that decays with time [33]. IRS was initially parametrized in [33], and represents the Actellic 300CS insecticide, such that in the model, after deploying IRS, the availability of a protected host to mosquito is reduced by 0.28, the probability of a mosquito successfully biting a chosen protected host is decreased by 0.23, and the probability of a mosquito successfully escaping from a protected host after feeding is decreased by 0.38.

To fit the simulations to geographic-specific transmission settings, the model requires an estimate of the EIR. To estimate region specific EIRs, the model was calibrated to *Pf*PR_2 − 10_ estimates [8], by selecting through a grid search the simulated EIR that maximizes a Gaussian likelihood function. Uncertainty in prevalence estimates is propagated by adapting the Monte-Carlo profile likelihood intervals [34]. Further details about the calibration method can be found in Lemant et al. (preprint) [35]. Calibration was performed on the historical *Pf*PR_2 − 10_ time-series between years 2005 and 2021, simulation fits and historical intervention coverages can be found in the Supplementary materials.

Simulations for each admin-1 region for Kenya, Mozambique, and Benin, were performed with mean EIR, and upper and lower uncertainty intervals resulting from the calibration. Historical interventions were implemented from 2000 to 2022, starting in 2023 bednet coverage remained constant (with the same coverage than 2022) and an incremental increase of effective treatment levels was implemented to reach 60% coverage in 2026, after which it remained constant. Simulations were repeated 10 times and ran up to the year 2031. Simulation inputs can be found in the *Additional File 1*.

### Impact estimates

Three metrics of impact associated with increase in case management coverage were computed. Firstly, the impact on prevalence was computed as the relative prevalence reduction, $\:100\:*\frac{PfP{R}_{x,baseline}-PfP{R}_{x,CM}}{PfP{R}_{x,baseline}}$, with subscript *baseline* indicating the prevalence in simulations without any increase in case management coverage (counterfactual), *CM* indicating the prevalence in simulations with an increase in effective treatment coverage, and subscript *x* indicating the age group for which prevalence was estimated. *x* refers to either the population of children under 5 years old or the entire population. The impact was estimated in 2031, 5 years after access to treatment had increased.

Next, impact on clinical cases was computed as the cumulative cases averted from the start of case management increase in 2023 to 5 years after case management increase, thus in 2031. Cases averted indicate the difference between cumulative number of cases in simulations without any case management increase (counterfactual) versus the number of cases for simulations with increased effective treatment coverage. Both total number of clinical cases averted, and severe clinical cases averted, were computed.

Lastly, to estimate the proportion of population living in different malaria risk areas, administrative regions in Benin and Mozambique were categorized as either lower risk (*Pf*PR below 0.15), medium risk (*Pf*PR between 0.15 and 0.3) and higher risk (*Pf*PR above 0.3) for children under five (*Pf*PR_0 − 5_) and for the entire population (*Pf*PR_0 − 100_). As the within-country prevalence in Kenya was much lower, administrative regions were categorized as regions with *Pf*PR below or above 0.01 for that country. For each age group, the proportion of population per given age group living in each malaria risk category (from the population estimates for each administrative region) was recorded, and the differences with and without improved effective access to care was computed.

## Results

### An increase in case management leads to prevalence reduction, across transmission intensities

**Fig. 1:**
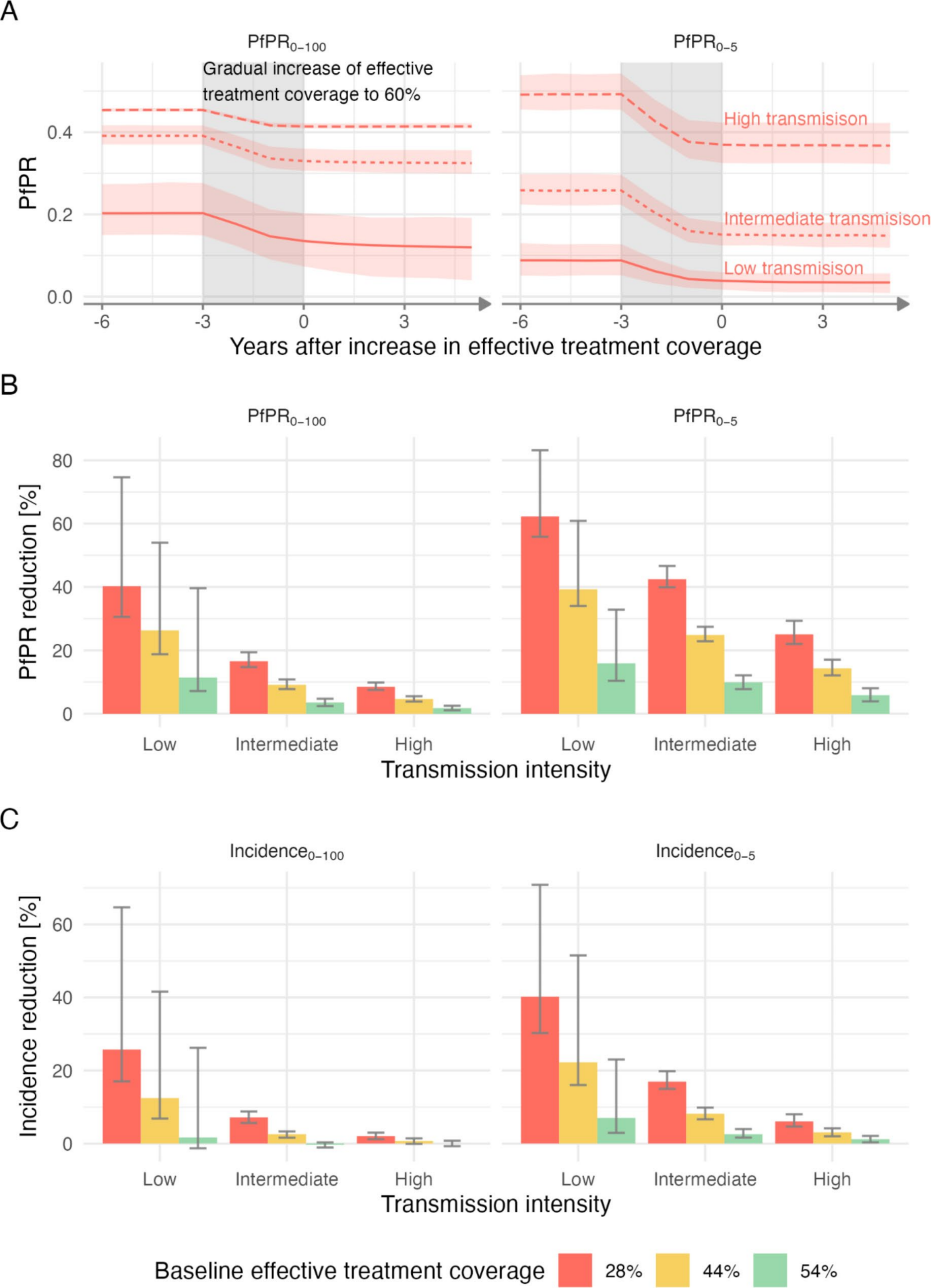
Increased case management leads to prevalence and incidence reduction across hypothetical settings. (**A**) Prevalence levels over 11 years of simulation time, across high transmission settings (EIR between 32–320), intermediate transmission (EIR between 8–32), and low transmission (EIR between 0.5–8), with an increase in effectively treated cases from 28–60% (gradual increase over 3 years, grey shaded area on the plots). (**B**) Relative reduction in prevalence 5 years after case management increased to 60%. (**C**) Relative reduction in incidence 5 years after case management increased to 60%. B-C) Relative reductions are shown for different transmission intensities (x-axis) and three different baseline coverage of effective treatment (colours). Panels in A-C) show outputs in total population in the left panels and in children under 5 years old in the right panels. Mean and interquartile range are shown for 10 seeds for each EIR level (EIR levels from 0.5 to 320 with 0.5 step) for prevalence and relative prevalence and incidence reduction

**Fig. 2:**
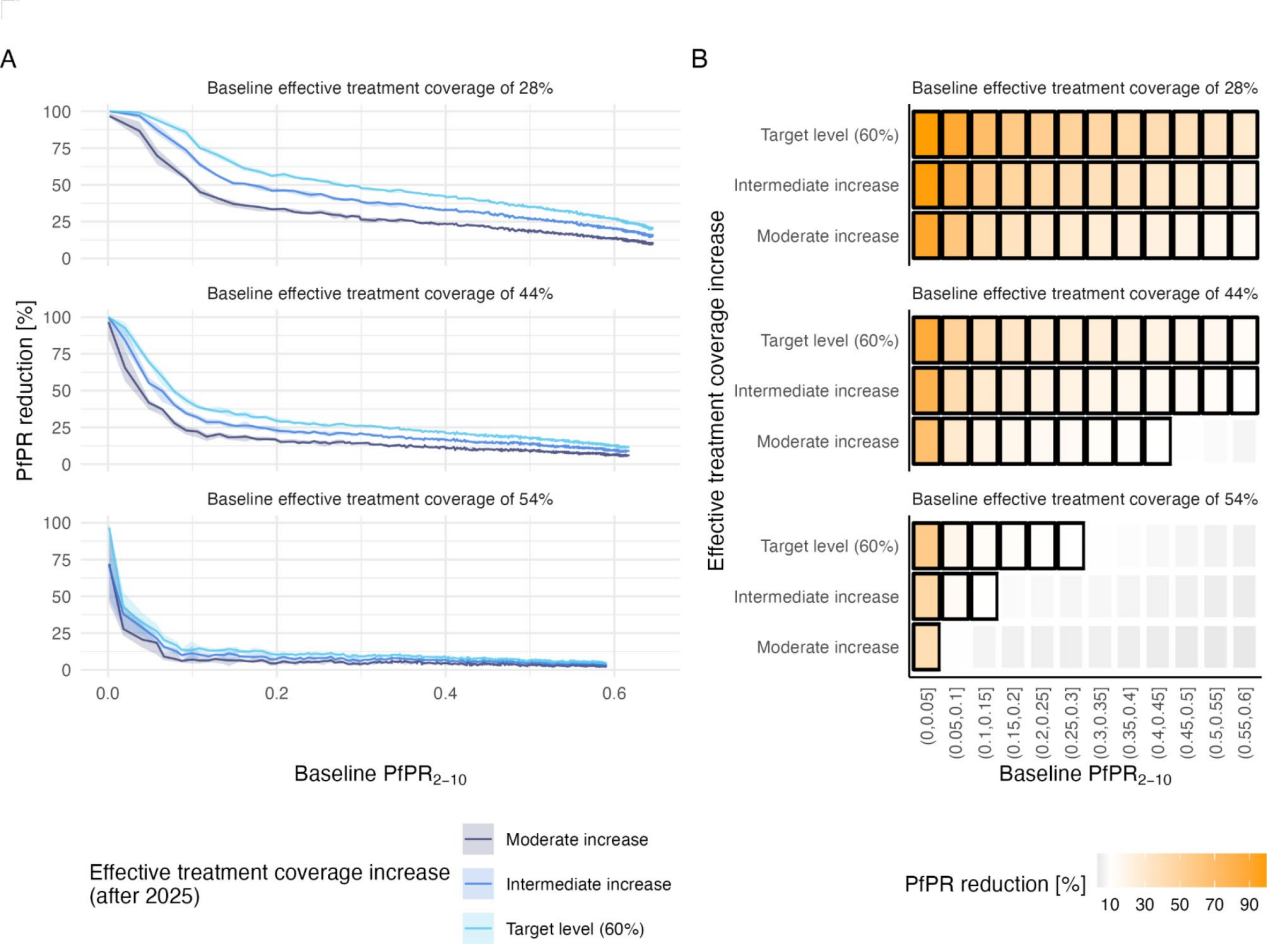
Increasing effective treatment in hypothetical settings shows greatest impact at low transmission settings and low baseline effective treatment coverages. (**A**) Relative reduction in prevalence in children under 5 years old (PfPR_0 − 5_) 5 years after case management increased. Reduction in prevalence is shown for varying transmission intensities (i.e. estimated PfPR_2 − 10_ when case management remains unchanged). Colours indicate different coverages of effective treatment after case management increase, and panels top to bottom indicate increasing baseline coverages of effective treatment. Lines represent the mean and shades the interquantile range across 10 seeds. (**B**) Impact, as relative reduction in PfPR_0 − 5_ (tiles), in function of baseline transmission intensity (PfPR_2 − 10_, x-axis), levels of effective treatment coverages increase (y-axis), and baseline coverages of effective treatment (panels top to bottom). Low to no impact are shown in grey shades and increasing impact in orange. Settings with impact above 10% are highlighted in black squares. In A) and B), increase of effective treatment is either target level (effective coverage of 60%), moderate relative increase of 50% from baseline coverage to the target level (Moderate increase), or intermediate relative increase of 75% from baseline coverage to the target level (Target increase)

In the simulations of hypothetical settings spanning a wide range of transmission intensities, improving effective treatment coverage from 28 to 60% - that is, improving from the 25th percentile to the best coverage currently estimated in Africa – resulted in malaria prevalence reduction in children under 5 years old and in the total population (Fig. 1A*)*. In lower transmission settings (EIR < 8, *Pf*PR_2 − 10_ < 0.25), *Pf*PR_0 − 5_ was reduced by 62.27% (interquartile range: [55.85–83.16]) and population level *Pf*PR was reduced by 40.24% (interquartile range: [30.55–74.62]). In intermediate transmission settings (EIR between 8 and 32, *Pf*PR_2 − 10_ between 0.25 and 0.45) the reduction was 42.47% for *Pf*PR_0 − 5_ (interquartile range: [39.91–46.63]) and 16.6% for population level *Pf*PR (interquartile range : [14.73–19.4]); and in high transmission settings (EIR between 32 and 320, *Pf*PR_2 − 10_ between 0.45 and 0.65) the reduction was 25.06% for *Pf*PR_0 − 5_ (interquartile range: [22.02–29.34]) and 8.51% for population level *Pf*PR (interquartile range : [7.52–9.86]) (Fig. 1B and Table S1). Relative reduction in incidence was also observed in the simulations, although at lower magnitude (Fig. 1C). In lower transmission settings (EIR < 8), incidence in young children was reduced by 40.2% (interquartile range: [30.32–70.82]) and population level incidence was reduced by 25.74% (interquartile range: [17–64.67]). In intermediate transmission settings (EIR between 8 and 32) the reduction in incidence was 16.95% in young children (interquartile range: [14.97–19.79]) and 7.17% for population level incidence (interquartile range : [5.62–8.78]); and in high transmission settings (EIR between 32 and 320) the reduction was 6.08% for young children (interquartile range : [4.66–8.02]) and 2.05% for population level incidence (interquartile range : [1.21–2.99]) (Fig. 1C and Table S2). Considerable impact was also observed both for higher baseline effective treatment levels and smaller case management increases, especially on prevalence (Figs. 1B-C and 2A, and Table S1-2and Figure S1). By increasing access to care, more clinical malaria cases are treated, thus shortening the length of treated infections. This leads to a reduction in the infectiousness of humans to mosquitoes in the population, which explains the indirect effect of access to care on transmission intensity.

Although the impact of expanded access to care was observed across transmission settings, the greatest relative prevalence reduction was observed in low transmission settings. The inverse relationship between transmission intensity and the impact of case management increase was nonlinear (Fig. 2A and Fig S2). To further investigate in which settings prevalence reduction was most consequential, the impact on prevalence was plotted against baseline PfPR_2 − 10_, and settings resulting in a 10% or higher prevalence reduction were identified (Fig. 2B, and Fig S2). More than 10% reduction in prevalence in children under 5 years old was observed across the investigated prevalence range (PfPR_0 − 5_ below 0.6) as a result of increasing effective access to treatment from 28 to 44% (Moderate increase) of higher. Similarly, more than 10% reduction in prevalence in children under 5 years old was reached across the prevalence range as a result of increasing effective access to treatment from 44 to 52% (Moderate increase) or higher. For higher initial effective treatment coverages of 54%, 10% of higher prevalence reduction was observed only for lower PfPR_2 − 10_ levels. In the total population, the magnitude of prevalence reduction was lower Fig S2. Transmission, defined as the population level infectiousness to biting mosquitos, is not easily assessed in the field but was estimated in the models. Population level infectiousness is defined as the sum of individual level infectiousness, which results from the host’s gametocyte density, body surface area, and availability to mosquitoes [27, 36]. Like prevalence, the relative reduction in transmission was observed following increased effective treatment, with more than 10% reduction in children under 5 years in settings of PfPR_2 − 10_ below 0.4 when increasing effective treatment from 28% to target level of 60% (Figure S3).

### An increase in case management leads to averted clinical cases

For the same observed prevalence, a higher treatment rate corresponds to a higher EIR compared to a setting with lower treatment rates, as the EIR-prevalence relationship is influenced by treatment rates (*Figure S4*, and described in Penny et al. 2015 [37]). Higher EIRs increase population immunity, which in turn leads to a higher relative proportion of cases in younger age groups where exposure-driven natural immunity has not yet developed. Consequently, for the same prevalence levels — especially at higher prevalence levels — we observe fewer overall cases but a greater concentration of cases in the 0–5 age group when case management is more effective (*Figure S5*). In addition, natural immunity prevents patent parasitemia from progressing to symptomatic disease. As a result, an increase in prevalence does not lead to a proportional increase in incidence (*Figure S5-A*). Conversely, a reduction in prevalence due to increased effective treatment does not produce a reduction in incidence of the same magnitude, especially in higher transmission settings (*Figure S6*).

Increasing effective access to treatment resulted in fewer malaria cases. Highest averted clinical cases were observed in the simulations in low transmission settings (EIR < 8) and with low effective treatment coverages prior increase (28%) (Figure S8 and Table S3). In those settings, simulations resulted in 2’683.11 clinical cases averted per 1’000 children under 5 years old (interquartile range: [2367.42–2872.13]) and 1’895.22 clinical cases averted per 1’000 population (interquartile range: [1549.94–2498.5]), cumulative over 5 years following the increase in effective treatment. The model also estimated incidence of severe cases, as a result of very high parasitemia or co-infection [28]. The number of severe cases averted in low transmission settings in children under 5 years old was 98.74 per 1’000 (interquartile range: [71.81–113.3]) and 32.89 per 1’000 in total population (interquartile range: [27.69–36.94]) Table S3.

The relationship between cases averted and underlying prevalence was nonlinear, with greatest impact in total population peaking at low transmission intensities (PfPR_2 − 10_ around 0.05) and in children under 5 years old peaking around PfPR_2 − 10_ = 0.2 (*Figure S9*). In the model, uncomplicated clinical cases in the total population followed a steep increase with transmission in very low transmission settings (PfPR_2 − 10_ below 0.1) and then reached a plateau (*Figure S10*) as a result of higher population level blood-stage immunity – which explains why most impact in case reduction was observed in this lower PfPR_2 − 10_ range. While our results indicate the possibility of greater marginal impact on prevalence and case reduction when increasing effective coverage from lower rather than higher baseline effective treatment coverages (*Figure S11*), additional studies are required to confirm this conclusion.

### Increasing case management leads to a reduction in prevalence and burden in transmission settings resembling Benin, Mozambique, and Kenya

**Fig. 3:**
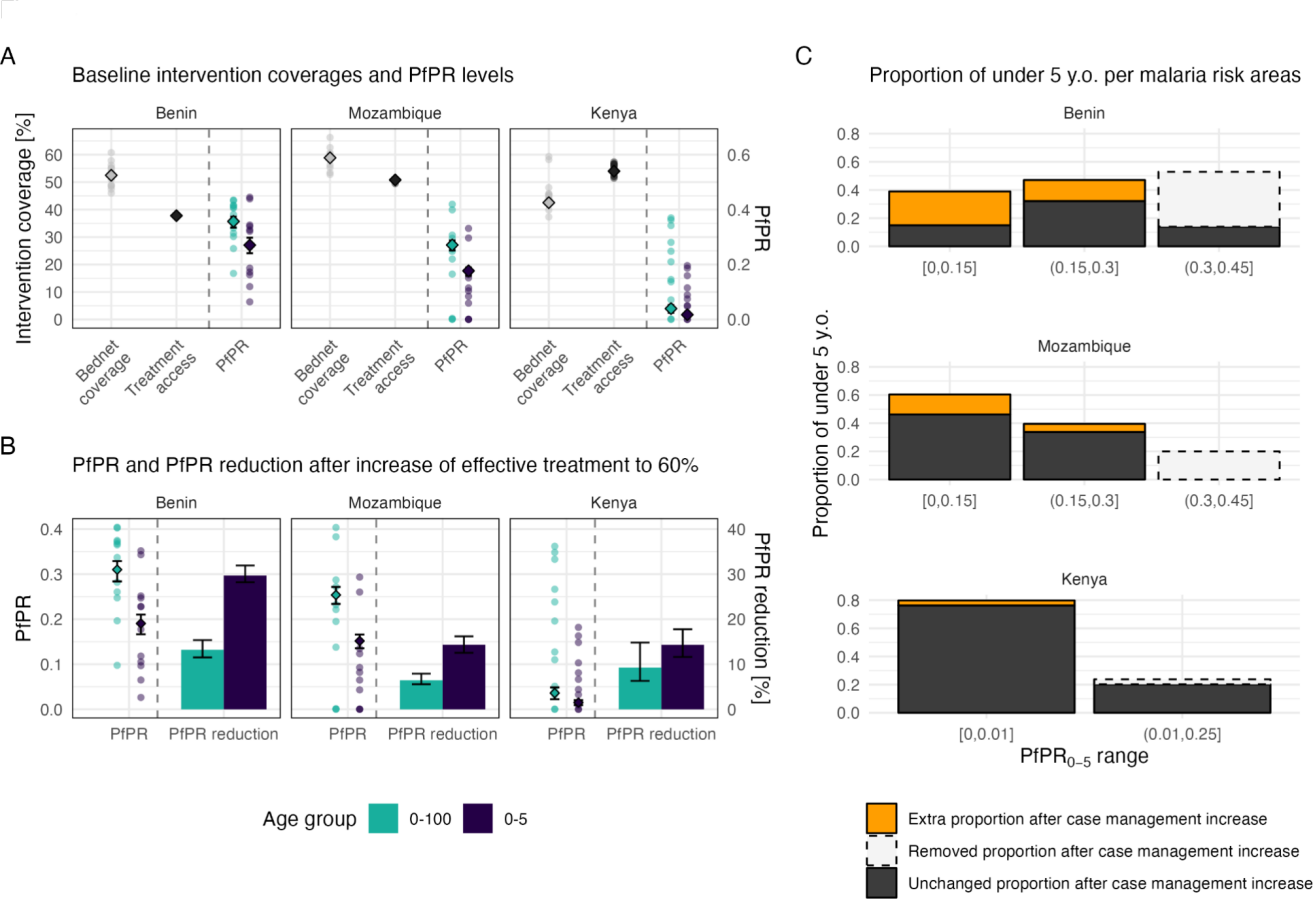
Malaria prevalence is reduced following an increase of case management to 60% coverage in Kenya, Mozambique, and Benin. (**A**) Bednet coverage, effective access to treatment and prevalence estimates for total population (light green), and children under 5 years old (purple), are shown in 2035, when effective treatment levels remain unchanged. Points represent mean estimates for each administrative unit within countries, and diamonds indicate the national level average (with minimum, and maximum across 10 seeds in the error bars). (**B**) Prevalence estimates for total population (light green), and children under 5 years old (purple), are shown in 2035, when effective treatment levels increased to 60%. Points represent mean estimates for each administrative unit within countries, and diamonds indicate the national level average (with minimum, and maximum across 10 seeds in the error bars). Relative prevalence reduction in both age-groups at national level are indicated by bars. (**C**) Proportion of children under 5 living in regions with lower malaria risk (PfPR_0 − 5_ less than 0.15), intermediate risk (PfPR0-5 between 0.15 and 0.3), and higher risk for malaria (PfPR_0 − 5_ greater than 0.3). Solid bars indicate the total proportion of the children under 5 years old living in each area (x-axis) in 2031 – with the orange bars the proportion of children who were not in given area prior case management increase – and dashed bars indicate the proportion of children under 5 years old that was living in given area prior case management increase but do no longer live in given area after case management increase. Mean across 10 seeds is shown

Reduction in prevalence and malaria burden can be achieved in countries of varying transmission context. Kenya, Mozambique, and Benin, which are representative of the subnational distribution of *Pf*PR_2 − 10_ and effective access coverage observed across African countries, show variable *Pf*PR_2 − 10_ and effective treatment coverage levels (Fig. 3A and Table S4). The average national *Pf*PR_2 − 10_ in Kenya, Mozambique, and Benin was estimated at 0.026 (minimum and maximum [0.017–0.036]), 0.24 (minimum and maximum [0.22–0.27]), and 0.35 (minimum and maximum [0.31–0.39]), respectively. Baseline effective treatment coverage was 53.86% in Kenya, 50.8% in Mozambique, and 37.78% in Benin, and bednet coverage was estimated at 42.65% in Kenya, 58.83% in Mozambique, and 52.46% in Benin. Intervention coverages were fairly consistent across administrative regions within each country, but prevalence varied considerably between administrative regions, with *Pf*PR_2 − 10_ as low as 0.0006, 0.0035, and 0.083, and as high as 0.29, 0.45, and 0.55, in Kenya, Mozambique, and Benin, respectively.

Prevalence reduction following an increase of effective treatment coverage to 60% was expected in all three countries. Impact was highest in Benin – where baseline case management was lowest compared to the two other investigated countries – with an estimated reduction in national level *Pf*PR_2 − 10_ of 24.99% (minimum and maximum [23.90 − 27.42]). A lower impact was observed in Mozambique, with an estimated reduction in *Pf*PR_2 − 10_ of 11.84% (minimum and maximum [11.04–12.24]), and in Kenya where baseline access to effective treatment was highest and, as such, with the lowest case management increase, the reduction in prevalence was modest (Fig. 3A-B, and Table S4).

By reducing prevalence, improving case management led to fewer populations living in high-risk areas for malaria. By categorizing each administrative region in each country by their *Pf*PR_0 − 5_ level, regions were assigned to lower risk (*Pf*PR_0 − 5_ < 0.15), moderate risk (*Pf*PR_0 − 5_ between 0.15 and 0.3), and higher risk (*Pf*PR_0 − 5_ between 0.3 and 0.45). The proportion of children under 5 years old of regions in each risk category is shown in Fig. 3C. An estimated 39% of children under 5 years old in Benin and 20% in Mozambique would no longer live in higher risk areas with access to effective treatment increasing to 60%. The same pattern was observed across the entire population, with an estimated 21% and 7.6% of the total population that would move from high-risk area to lower risk area in Benin and Mozambique, respectively (*Figure S11*). In Kenya where most of the population lives in areas with prevalence below 0.15, and case management is fairly high (53.9%), 0.39% of children moved to lower-risk areas.

Subnational heterogeneity in prevalence, and to some extent heterogeneity in effective treatment coverage, resulted in subnational differences in the impact of improved effective access to treatment. The inverse relationship between impact and transmission intensity predicted in the hypothetical settings was confirmed in the subnational simulations, where highest impact in relative prevalence reduction was observed in administrative units of lowest *Pf*PR_2 − 10_ (*Figure S12*). Uncertainties in the model estimates were generally higher in very low transmission areas, as for example in Turkana, Bungoma, Mombasana, Kwale, and Kakamega in Kenya, and in Maputo, Maputo City, and Gaza in Mozambique. This is both because modeling low transmission settings in OpenMalaria is less certain and because of the stochastic nature of the model. For the administrative regions cited above, interruption of transmission was reached in some iterations of the simulation (i.e. relative reduction in prevalence of 100%).

### Increasing case management leads to a moderate reduction in incidence in countries like Benin and Mozambique

**Fig. 4:**
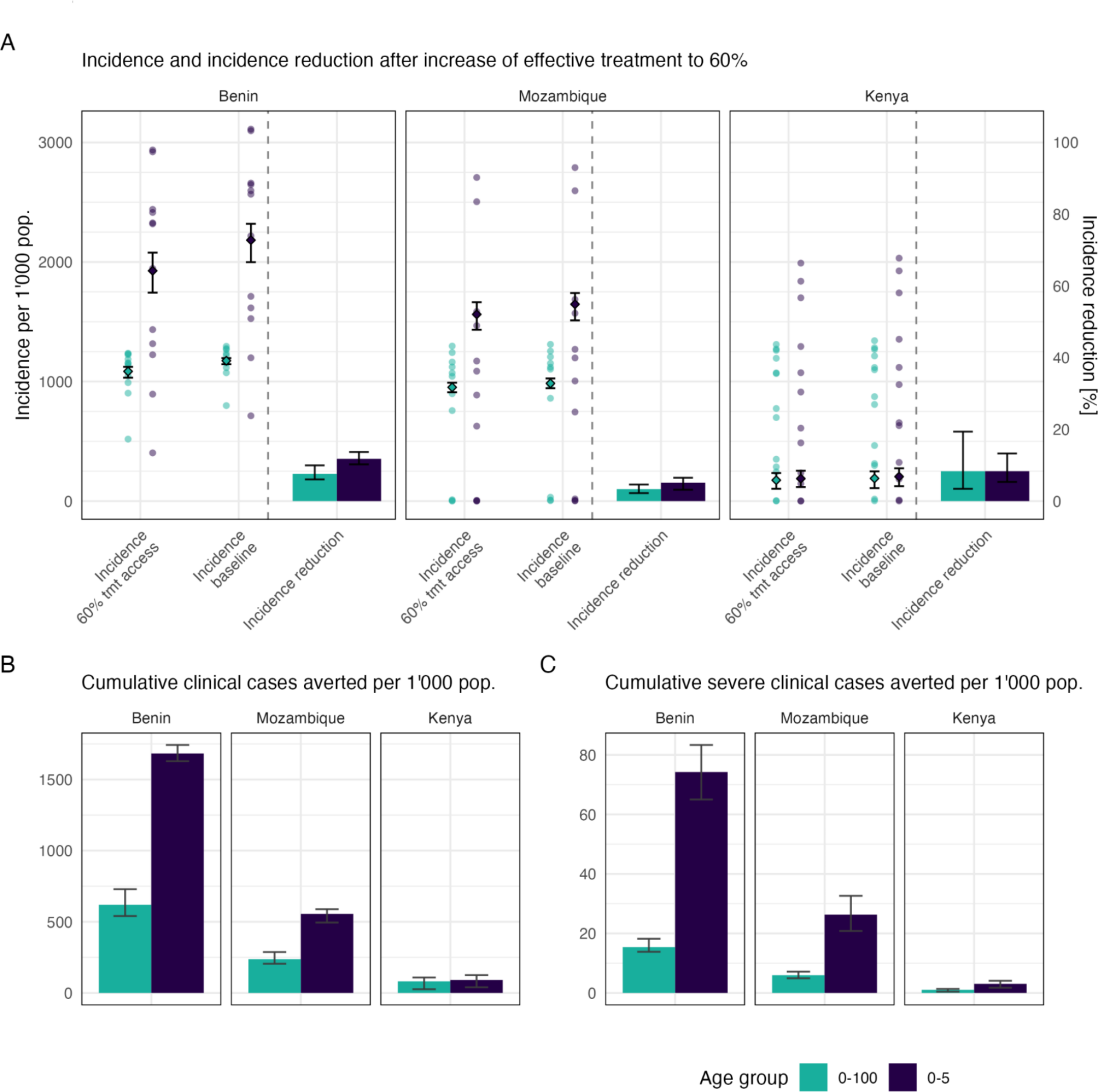
Moderate incidence reduction following an increase of case management to 60% coverage in Kenya, Mozambique, and Benin. (**A**) Incidence and incidence reduction after increase of effective treatment to 60%. Incidence after effective coverage increased to 60% (Incidence 60% tmt access), and without increase in effective treatment coverage (Incidence baseline) are indicated for each administrative unit within countries by points and the national level average by diamonds (with minimum, and maximum across 10 seeds). Relative reduction in incidence 5 years after treatment coverage increase are indicated for national level estimates by bars. (**B**) Cumulative clinical cases averted, and (**C**) cumulative severe clinical cases averted, per 1’000 population in total population (green), and in children under 5 years old (purple). Cases averted computed as the cumulative over 5 years from 2026 to 2031. In each plot A-C), mean, minimum, and maximum across 10 seeds are shown

Five years after increase of effective access to treatment, yearly incidence per 1’000 children under 5 years old was predicted to decrease from 2’182.72 [1’998.64–2’317.67] to 1’926.44 [1’744.02–2’078.47] in Benin, from 1’646.59 [1’512.02–1’740.23] to 1’561.97 [1’433.78–1’663.11] in Mozambique, and from 204.61 [124.48–274.50] to 188.02 [117.81–253.29] in Kenya (Fig. 4A and Table S5). Over the 5 years following the increase in effective treatment, an estimated 1’682.33 averted cases per 1’000 children under 5 years old (minimum and maximum [1’628.35–1’742.6]) and 74.24 averted severe cases per 1’000 children under 5 years old (minimum and maximum [65–83.33]) were observed in Benin, 555.58 averted cases per 1’000 (minimum and maximum [494.5–588.67]) and 12.65 averted severe cases per 1’000 children under 5 years old (minimum and maximum [9.47–18.73]) in Mozambique (Fig. 4B-C, and Table S6).

## Discussion

Treating uncomplicated malaria cases prevents severe outcomes and deaths, but the indirect effects of treatment on transmission have remained unclear due, in part, to the complexity of disentangling effects across simultaneous interventions in surveillance or other data sources. Using an agent-based model of malaria dynamics [19], we were able to investigate the potential impact on transmission of expanding access to effective treatment to 60% coverage, across transmission settings. Simulations indicated that better effective treatment coverage leads to prevalence and incidence reduction. Reduction in prevalence was observed across transmission intensities, with a nonlinear relationship between baseline prevalence levels and impact of case management. Impact was observed across the population but was highest in young children. Reduction in incidence was of lesser magnitude (1–35% in young children) than reduction in prevalence (5–58% in young children), especially in higher endemic settings where little to no impact on incidence was observed (1–5% in young children). Although in some of the simulations where prevalence was low, elimination was achieved by increasing case management to 60%, this might be an over-optimistic result. While sustained case management is crucial for elimination, the integration of active case detection and more aggressive vector control strategies is likely necessary as well [38, 39]. Nevertheless, a recent study on sub-national elimination in Myanmar showed that intensifying early diagnosis and treatment led to elimination in several villages [40], suggesting that an upscale in case management could be enough for elimination given the right context.

Simulations calibrated to resemble malaria transmission in Benin, Mozambique, and Kenya – countries where insecticide treated nets are already in place – indicated that increased case management would lead to a reduction in prevalence and burden in those three countries. In particular, we anticipate that for countries with transmission dynamics and effective treatment coverage levels similar to Benin and Mozambique, increasing effective treatment coverage would lead to substantial reduction in the proportion of young children living in areas with high malaria risk. For countries similar to Kenya with low prevalence and fairly high effective treatment coverage, improving treatment coverage would further reduce prevalence.

We investigated the potential impact of better access to care in three African countries, but the impact would be major across the continent. Global estimates of effective coverage of treatment indicated that in 2020, considerable African countries had suboptimal access to care, with 12 countries where effective access was below 30%, and 31 countries below 50% in 2020, out of the 40 countries [18]. As such, there is a need to sustainably improve access to effective care across Africa, and achieving a 60% coverage would have a tremendous impact, not only on burden, but also on transmission. Our analysis suggests that improving case management should be a tool to reduce transmission and prevalence in both low and high endemic areas. Indeed, when in low endemic areas it would be an additional step towards elimination, in higher endemic areas it would reduce malaria risk areas. Countries where current case management coverage is lowest should be prioritized given the biggest need and biggest impact predicted in this context.

The effect of treatment of uncomplicated malaria cases on transmission and burden may be several fold. By treating infections early, the average infection length in a population is reduced, leading to a decrease in the average infectivity in the population. This is particularly important in a pathogen like *Plasmodium falciparum*, where infections are known to be persistent or recurrent over months or years [41]. Recent studies indicate that the parasite’s commitment to gametocytes and thus transmission is smaller early in the infection compared to the longer chronic stages of the infections [42], thus treating infections early might have an even higher effect on transmission than anticipated in our study. Effective treatment may reduce incidence of uncomplicated cases and severe cases by its direct action of treating cases, and by reducing transmission, but the relative proportion of both effects could not be assessed here. While the current model does not include this feature, future modeling efforts that seek to disentangle the direct and indirect effects of case management on prevalence could provide valuable insights. In the current modelling study we found lesser impact of increasing effective treatment on incidence than prevalence, especially in higher endemic settings, which might be explained in part by the acquired immunity in the population protecting against clinical disease and gained through repeated exposure [43]. As such, impact of effective treatment might be underestimated if measured with routine reporting systems of clinical cases rather than community surveys of prevalence.

Our results align with previously published modeling work. For example, Okell et al. demonstrated a significant role of ACTs in transmission reduction strategies, especially in lower tranmission settings [44]. Similarly, a model by Gatton and Cheng suggests that interuption of transmission could be feasible in low to moderate transmission settings by treating symptomatic cases [45]. Another malaria transmission model showed that untreated infections, low treatment adherence leading to chronic untreated infection, and delayed treatment, all led to increased transmission [46]. Reducing the time between symptom onset and treatment through better access to care – for example by reducing the distance to the closest health facility [47] by increasing the number of health centers in a country – was shown to reduce the risk of severe malaria in a meta-analysis [48].

Increasing effective access to care through greater health systems performance can be tackled at different levels in the cascade of care [7, 49]. WHO recommends early diagnosis and treatment for effective case management [4]. Thus, better access to testing and treatment is essential [50, 51] and could be achieved by increasing the availability of diagnostics tests [52] and the number of trained workers to perform the tests and provide treatments [53, 54]. The provider compliance and the patient adherence to treatment regimen are other essential components impacting the effective coverage of care [7], and especially in adults appropriate care of malaria cases could be improved [55]. Finally, in order to maximise the drug’s efficacy, parasite resistance to drugs needs to be monitored and addressed rapidly if it arises [56]. Adherence, compliance, and drug quality has been found to vary substantially between countries [7], as such, improving effective treatment would involve identifying the source of inefficiency within this care cascade. In addition, better malaria case management would be achieved by strengthening health systems as a whole, thus moving from a malaria-focused approach towards a disease-holistic solution [57]. The current estimates show that the highest estimate of effective treatment was found in Uganda, of 61%. Previous analysis indicated that this country performed well across all components of the care cascade, in particular it showed high access to any provider (84%) and high adherence levels (93.9%) [7].

OpenMalaria has been previously developed and is discussed in detail elsewhere [19], but the model has some limitations relevant to this work. The model was mainly fitted to cross sectional surveys, limiting the certainty on implemented parasite dynamics. The absence of longitudinal data of malaria infections in endemic areas makes it challenging to accurately model immune dynamics. This difficulty extends to representing realistic – yet unknown – within-host malaria infection dynamics and understanding how changes in endemicity influence these dynamics through the acquisition or loss of immunity [26]. Assumptions on the length and intensity of infections in endemic areas directly affect population level infectivity, and thus impacts the computed effect of treatment on transmission. In addition, data for severe incidence in older age groups are lacking, and thus the model has been fitted to severe incidence in children under 10 years old [28]. As such, estimates of population level severe incidence remain uncertain. The model is calibrated primarily for moderate to high transmission settings, as a result, the accuracy of outcomes in low transmission settings is less certain. In addition, treatment seeking behavior for malaria infections in Africa are complex and remain uncertain [58], limiting both the estimates of effective access to care and the translation of improving access in the model to behavioral changes in country. The estimates of intervention coverages, effective access to treatment, and historical prevalence estimates from the Malaria Atlas Project to simulate sub-national settings in Benin, Mozambique, and Kenya, are modelled quantities, with areas of data paucity reflected in increased uncertainty.

Simulations focus on the effect of increasing effective treatment of symptomatic malaria cases across the population. This is only one aspect of improving case management, and further modeling studies could investigate the effect of expanding treatment by targeting vulnerable populations specifically, such as children under 5, or hard to reach communities. In addition, instead of investigating the effect of higher effective treatment, specifically modeling better treatment regimen resulting in greater compliance or higher cure rate, for example, deserves further investigation to understand its impact on transmission and malaria burden.

Although over the years there has been a shift towards greater investment into health system strengthening among major donors, such as the Global Fund or other Global Health Initiatives [59, 60], the potential to improve case management if investments are met are considerable across endemic countries. This work emphasizes the need to prioritize health system strengthening resulting in better effective treatment of malaria cases, which, in complement to vector control interventions, could achieve substantial burden reduction and transmission reduction across transmission settings.

## Electronic supplementary material

Below is the link to the electronic supplementary material.

Supplementary Material 1

Supplementary Material 2

## Data Availability

The input data used for the OpenMalaria simulations are found in the Additional file 1. Simulation outputs generated during the current study are available from the corresponding author, FC, upon reasonable request. OpenMalaria is an open-source C++ program available at https://github.com/SwissTPH/openmalaria/.Data generated by the Malaria Atlas Project are published on their website or available upon reasonable request.
