# Supplementary material for: Malaria treatment for prevention: a modelling study of the impact of routine case management on malaria prevalence and burden
Source: BMC Infect Dis. 2024 Nov 8;24:1267. doi: 10.1186/s12879-024-09912-x (PMC11549775; doi:10.1186/s12879-024-09912-x)
Supplement: Supplementary file 2 — Supplementary Material 2 [file 12879_2024_9912_MOESM2_ESM.pdf]

Supplementary Material for:

Malaria treatment for prevention: a modelling study of the impact of routine case management on malaria prevalence and burden

Authors

Flavia Camponovo[1,2], Aurélie Jeandron[1,2], Laura A Skrip[3], Monica Golumbeanu[1,2], Clara Champagne[1,2], Tasmin L. Symons[4,5], Mark Connell[4,5], Peter W. Gething[4,5], Theodoor Visser[6], Arnaud Le Menach[6], Justin M Cohen[6], Emilie Pothin[1,2]

[1]: Swiss Tropical and Public Health Institute, Basel, Switzerland

[2]: University of Basel, Basel, Switzerland

[3]: University of Liberia School of Public Health, Monrovia, Liberia

[4]:, Telethon Kids Institute, Perth Children's Hospital, Perth,, Australia

[5]: School of Population Health, Curtin University, Perth, Australia

[6]: Clinton Health Access Initiative, Boston MA, USA

corresponding author: flavia.camponovo@unibas.ch

Open Malaria calibration to Mozambique, Kenya, and Benin

The plots below show the prevalence estimates from OpenMalaria (blue line), and MAP (red dots) from 2005-2021 for all admin-1 units in each country. In addition, coverages of effective access to treatment (CM, light green), insecticide residual spraying (IRS, green), and insecticide treated nets (ITN, dark green) estimated by MAP are also indicated on each plot

Mozambique: observed and simulated historical prevalence

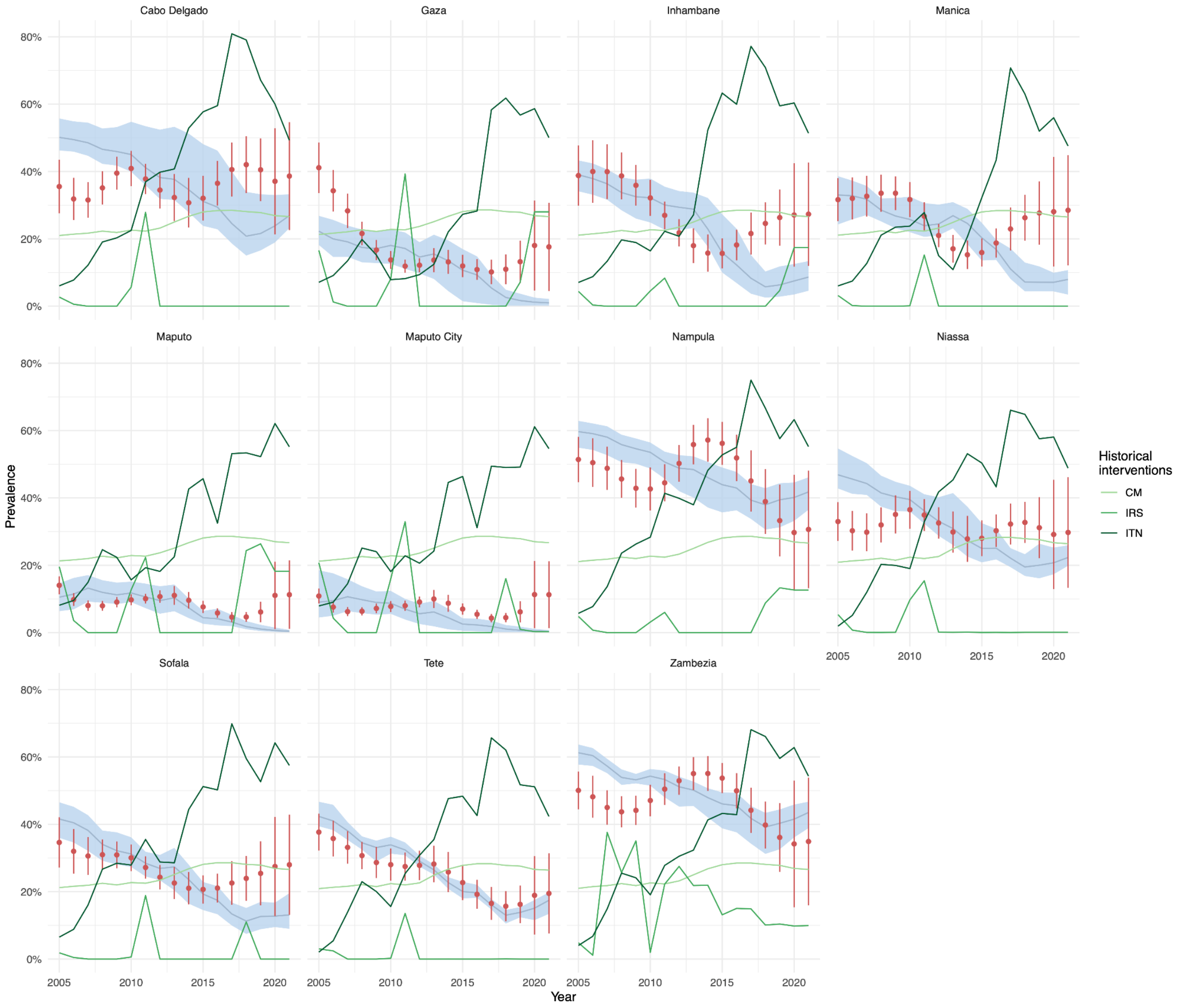

Kenya: observed and simulated historical prevalence

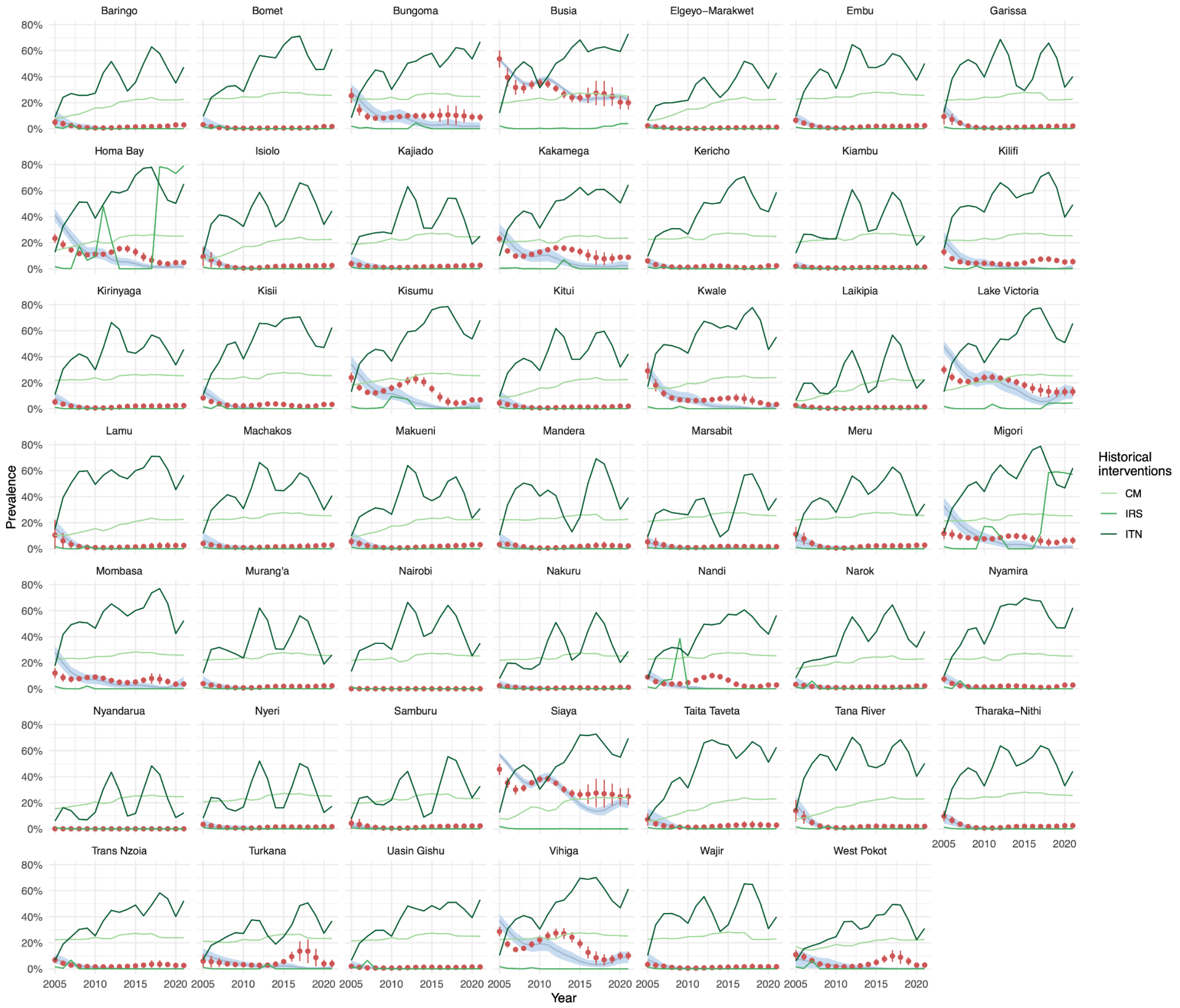

Benin: observed and simulated historical prevalence

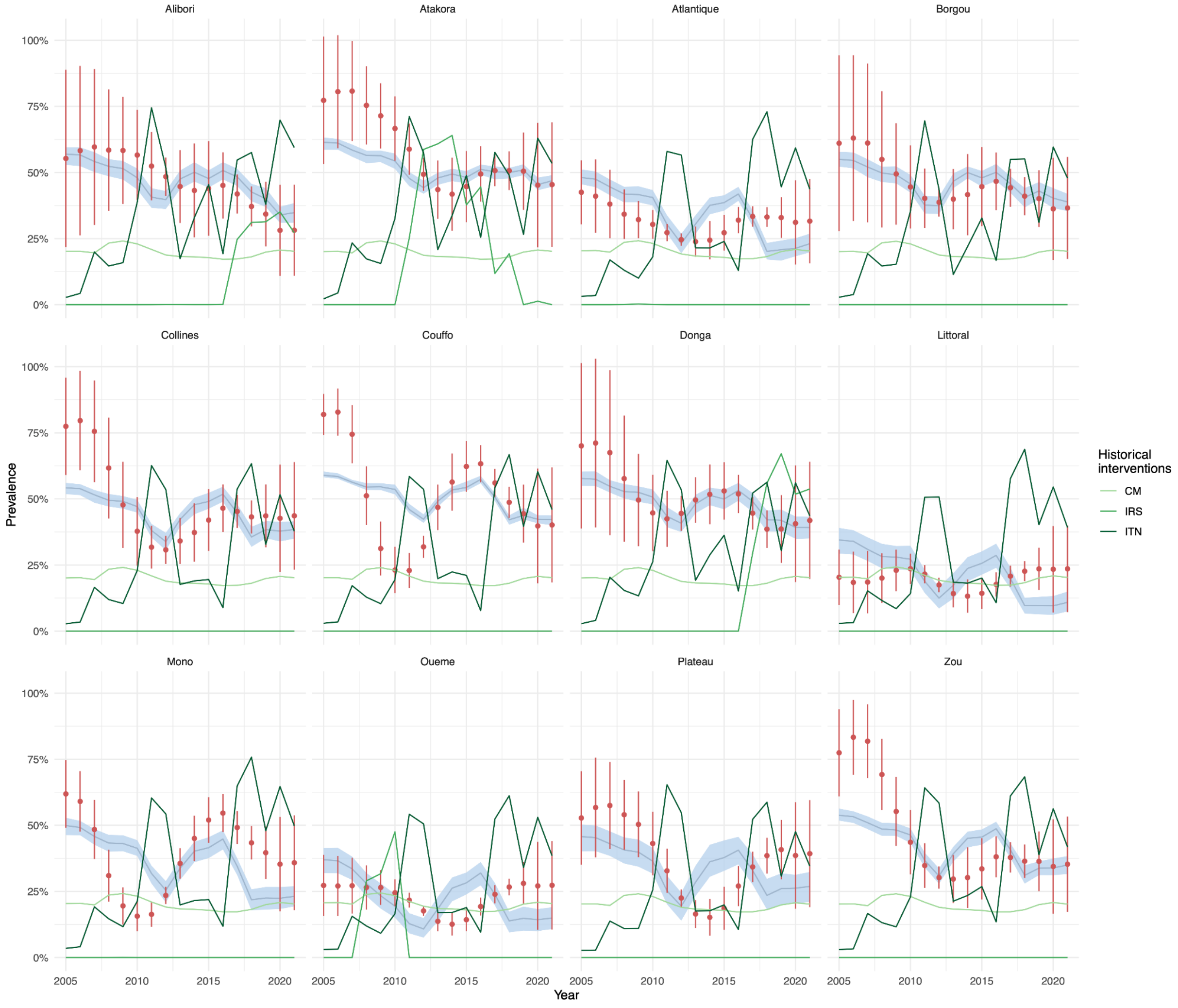

Supplementary figures

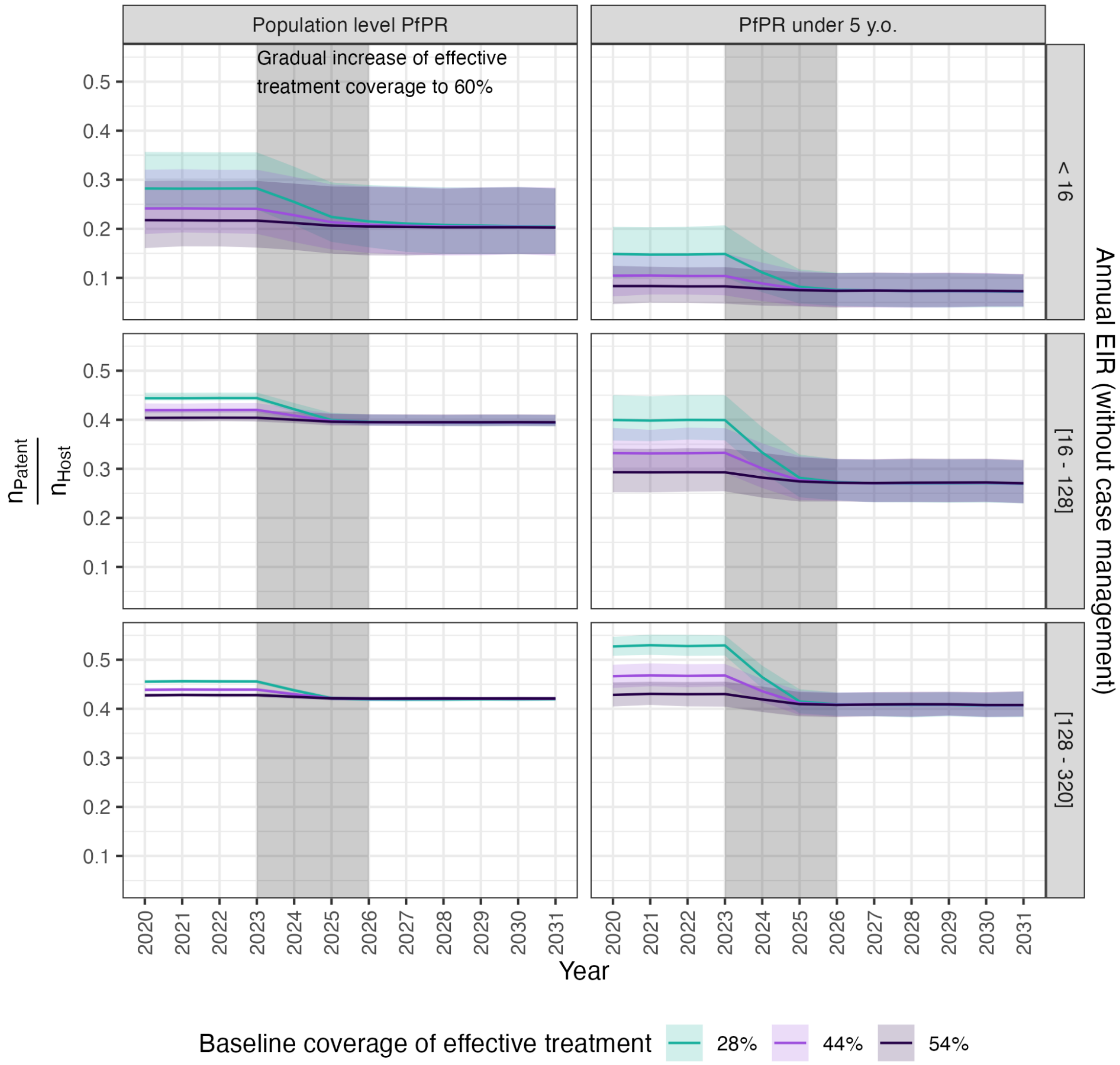

Figure S1. Increased case management leads to prevalence reduction. Prevalence levels from 2020 to 2031 for simulations across low transmission settings (top panels, EIR between 0.5–16), intermediate transmission (middle panels, EIR between 16–128), and high transmission settings (bottom panels, EIR between 128–320), with an increase in effective treatment coverage to 60%, starting from baseline effective treatment coverage of 28% (green), 44% (light purple), and 54% (dark purple) (gradual increase in years 2023 to 2026, indicated in grey shade). Mean and interquartile range are shown for 10 seeds for each EIR level (EIR levels from 0.5 to 320 with 0.5 step) for both prevalence in total population (left panels) and prevalence in children under 5 years old (right panels).

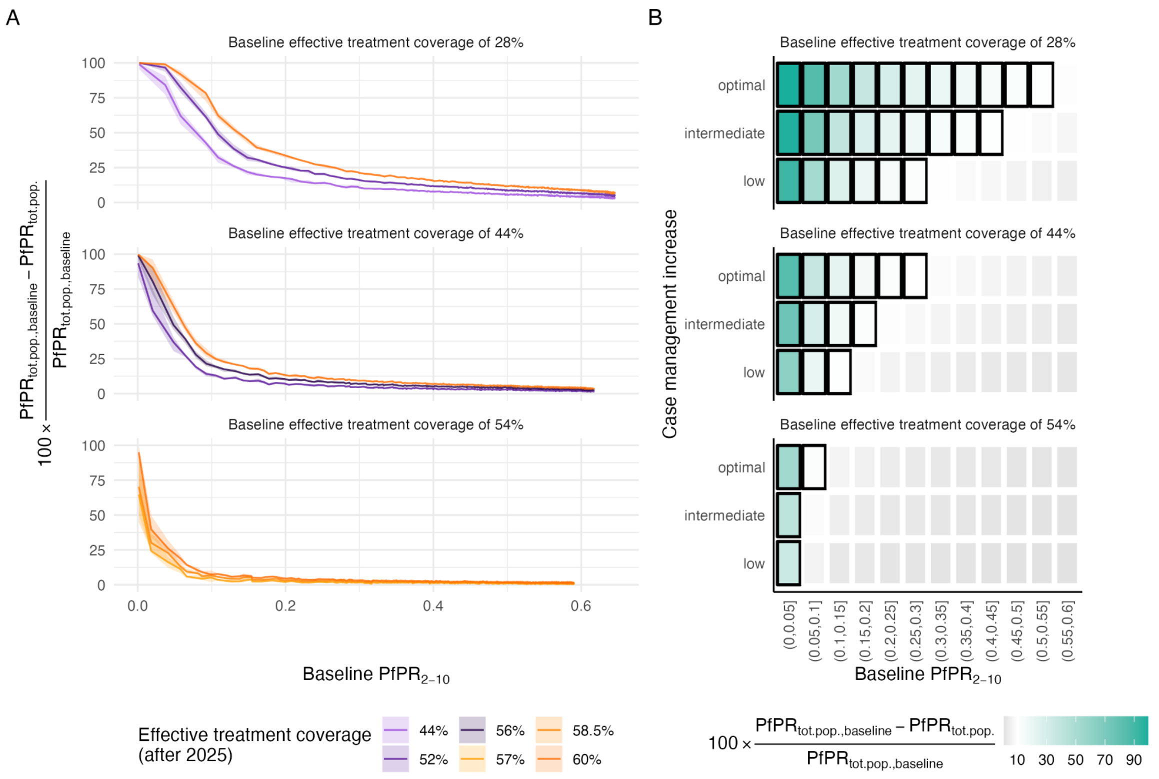

Figure S2. Prevalence reduction is highest at low transmission settings. A) Relative reduction in prevalence in total population ($PfPR_{tot.pop.}$), with $PfPR_{tot.pop.,baseline}$ prevalence with no change in case management and $PfPR_{tot.pop.}$ the prevalence after case management increase. Prevalence estimates are for the year 2031, 5 years after case management increased. Reduction in prevalence is shown for varying baseline $PfPR_{2\text{-}10}$ levels (i.e. estimated $PfPR_{2\text{-}10}$ in 2031 when case management remains unchanged). Coulours indicate different effective treatment coverages after case management increase and panels top to bottom indicate increasing baseline effective treatment coverages. Lines represent the mean and shades the interquantile range across 10 seeds. B) Impact, as relative reduction in $PfPR_{tot.pop.}$ (tiles), in function of baseline $PfPR_{2\text{-}10}$ (x-axis), increase in case management (y-axis), and baseline effective treatment coverage (panels top to bottom). Low to no impact are shown in grey shades, and increasing impact in green. Settings with impact above 10% are highlighted in black squares.

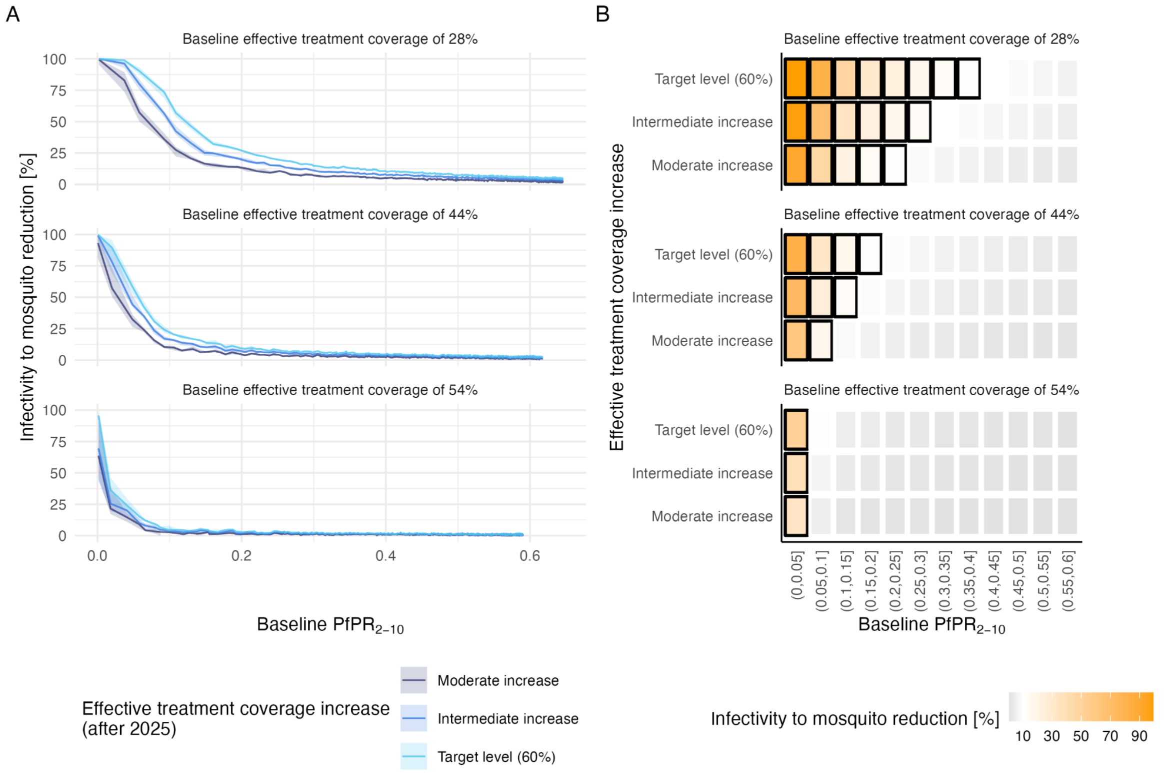

Figure S3 Transmission reduction is highest at low transmission settings. A) Relative reduction in infectivity to mosquito in total population. Infectivity is defined as total infectiousness of human population to mosquitoes. Prevalence estimates are for the year 2031, 5 years after case management increased. Reduction in prevalence is shown for varying baseline $PfPR_{2\text{-}10}$ levels (i.e. estimated $PfPR_{2\text{-}10}$ in 2031 when case management remains unchanged). Coulours indicate different effective treatment coverages after case management increase and panels top to bottom indicate increasing baseline effective treatment coverages. Lines represent the mean and shades the interquantile range across 10 seeds. B) Impact, as relative reduction in infectivity (tiles), in function of baseline $PfPR_{2\text{-}10}$ (x-axis), increase in case management (y-axis), and baseline effective treatment coverage (panels top to bottom). Low to no impact are shown in grey shades, and increasing impact in green. Settings with impact above 10% are highlighted in black squares.

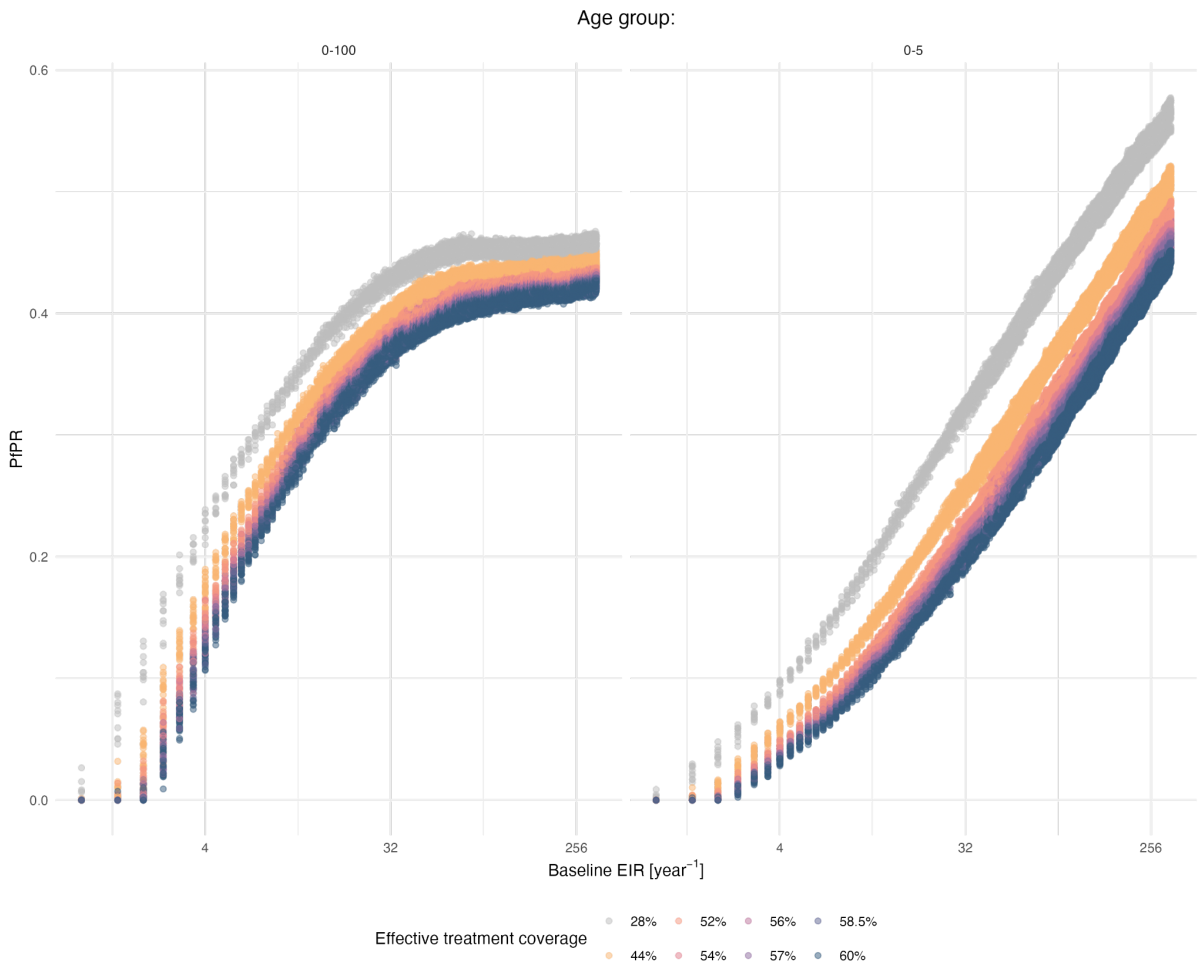

Figure S4. Prevalence in function of EIR and effective access to treatment. Estimated prevalence (y-axis) shown for the total population (left panel) and children under 5 years old (right panel), in function of the yearly EIR (x-axis). Colours indicate different levels of effective access to treatment. Results are shown as an average between years 2031-2035, and the mean across 10 seeds.

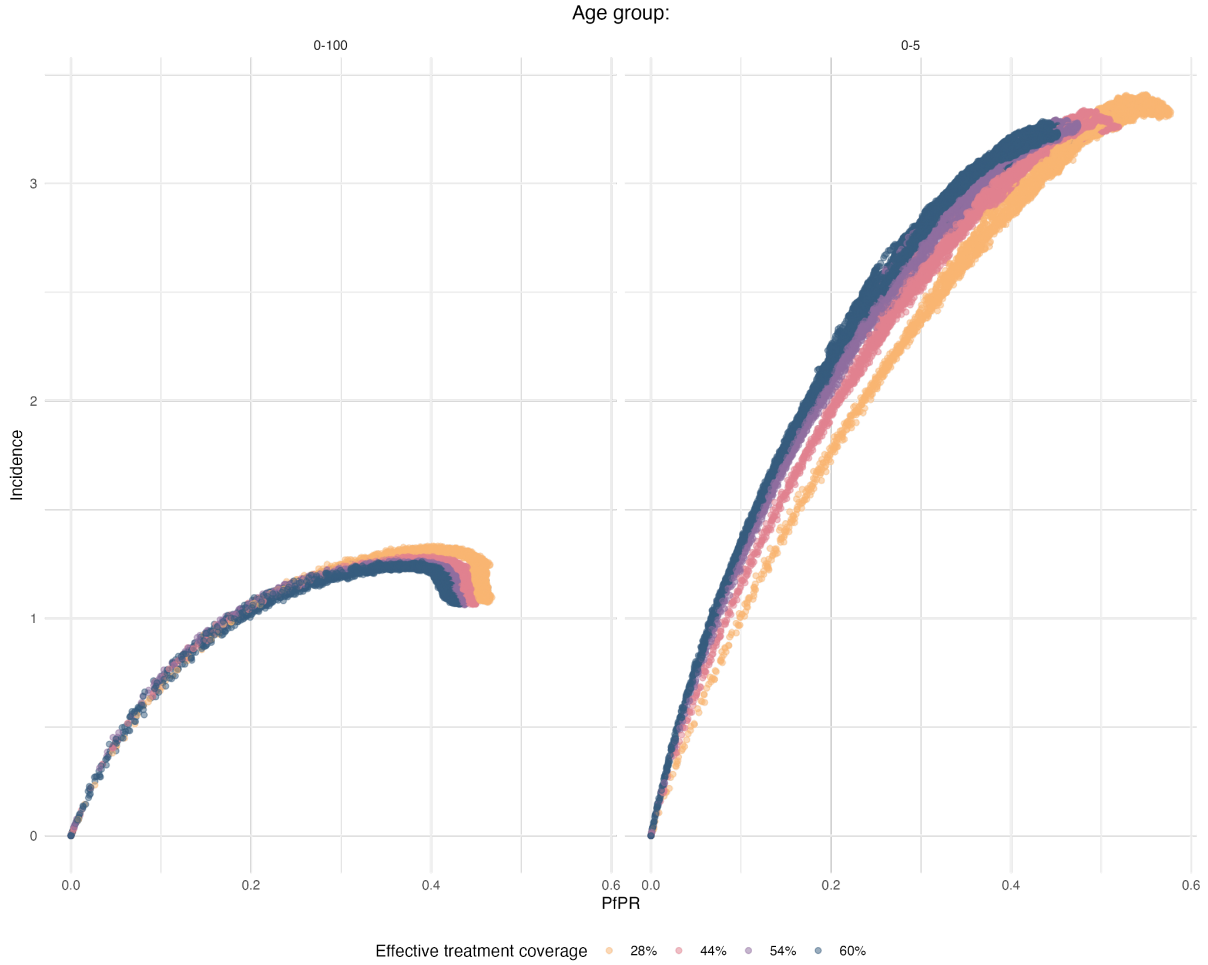

Figure S5. Relationship between prevalence (x-axis) and incidence (y-axis), for simulations with varying effective access to treatment (indicated by colours) Estimates for total population are shown in the left panel and children under 5 years old in the right panel. Results are shown as an average between years 2031-2035, and the mean across 10 seeds.

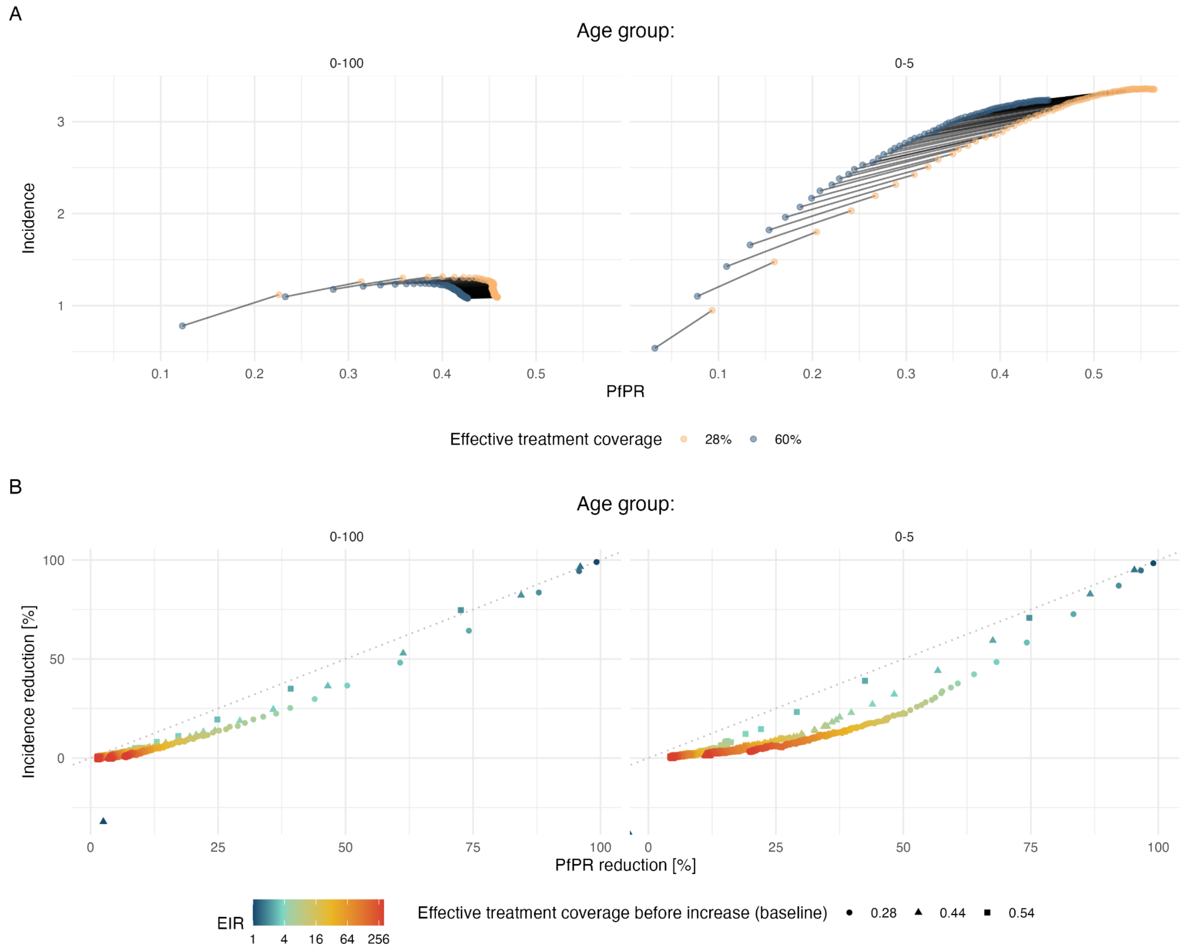

Figure S6. A) Relationship between Incidence (y-axis) and prevalence (x-axis), without increase in effective coverage (effective coverage at 28%, orange dots) compared to simulations with target effective treatment coverage (60%, blue dots). B) Relationship between relative incidence reduction (y-axis) and relative prevalence reduction (x-axis) after increasing to target effective treatment level of 60%, for varying levels of EIR (indicated by the colour gradient). Shapes indicated different levels of baseline effective treatment. Estimates for total population are shown in the left panels and children under 5 years old in the right panels. Results are shown as an average between years 2031-2035, and the mean across 10 seeds.

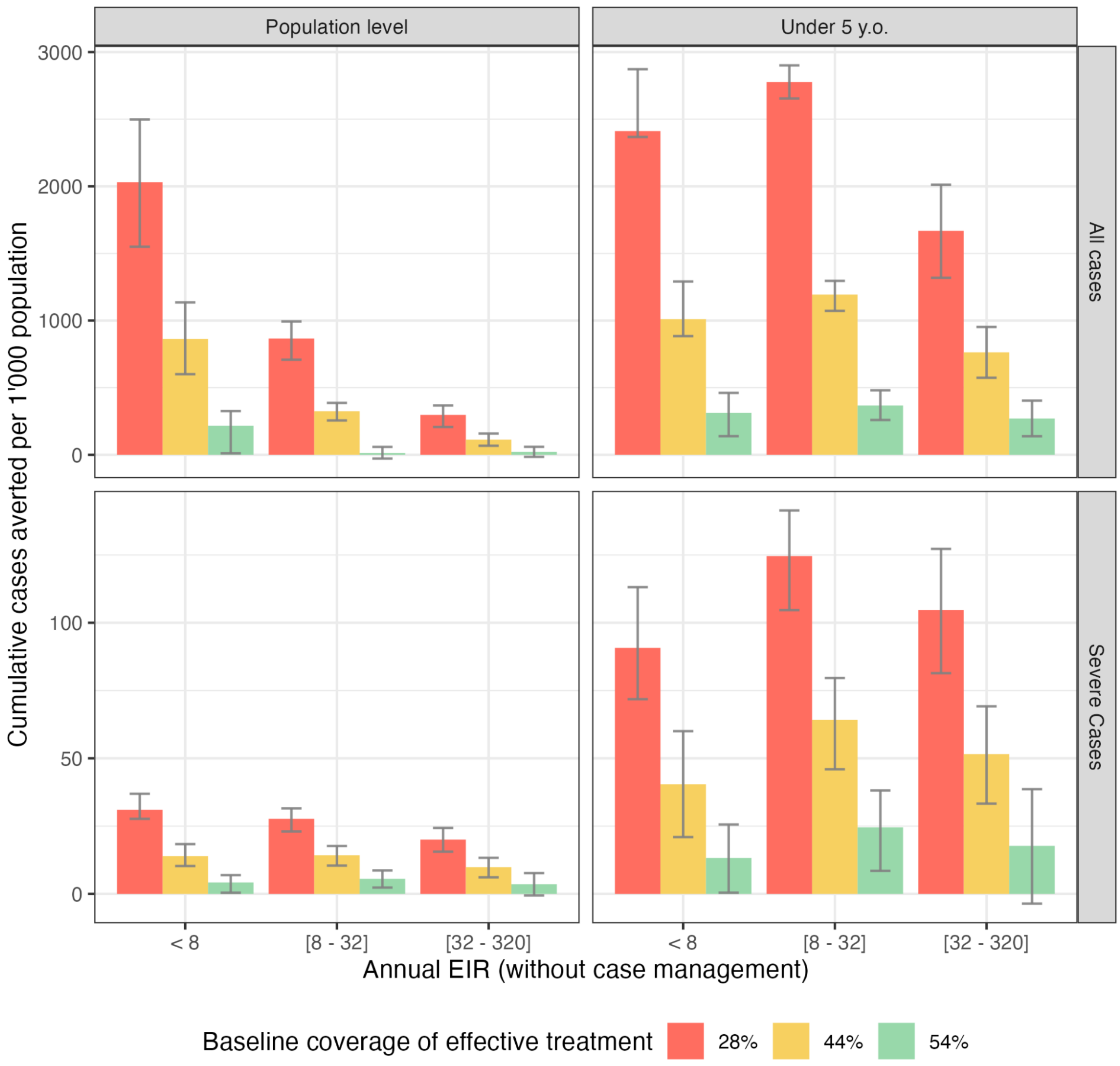

Figure S7. Increased case management leads to burden reduction. Cumulative cases averted per 1’000 population between 2023 and 2031 in total population (left panel) and in children under 5 (right panel). All clinical cases averted (top panels) and severe clinical cases averted (bottom panel) are shown when increasing effective treatment coverage to 60% starting from baseline effective treatment coverage of 28% (green), 44% (light purple), and 54% (dark purple), and the x-axis represent increasing EIR ranges. Mean and interquartile range are shown for 10 seeds for each EIR level (EIR levels from 0.5 to 320 with 0.5 step)

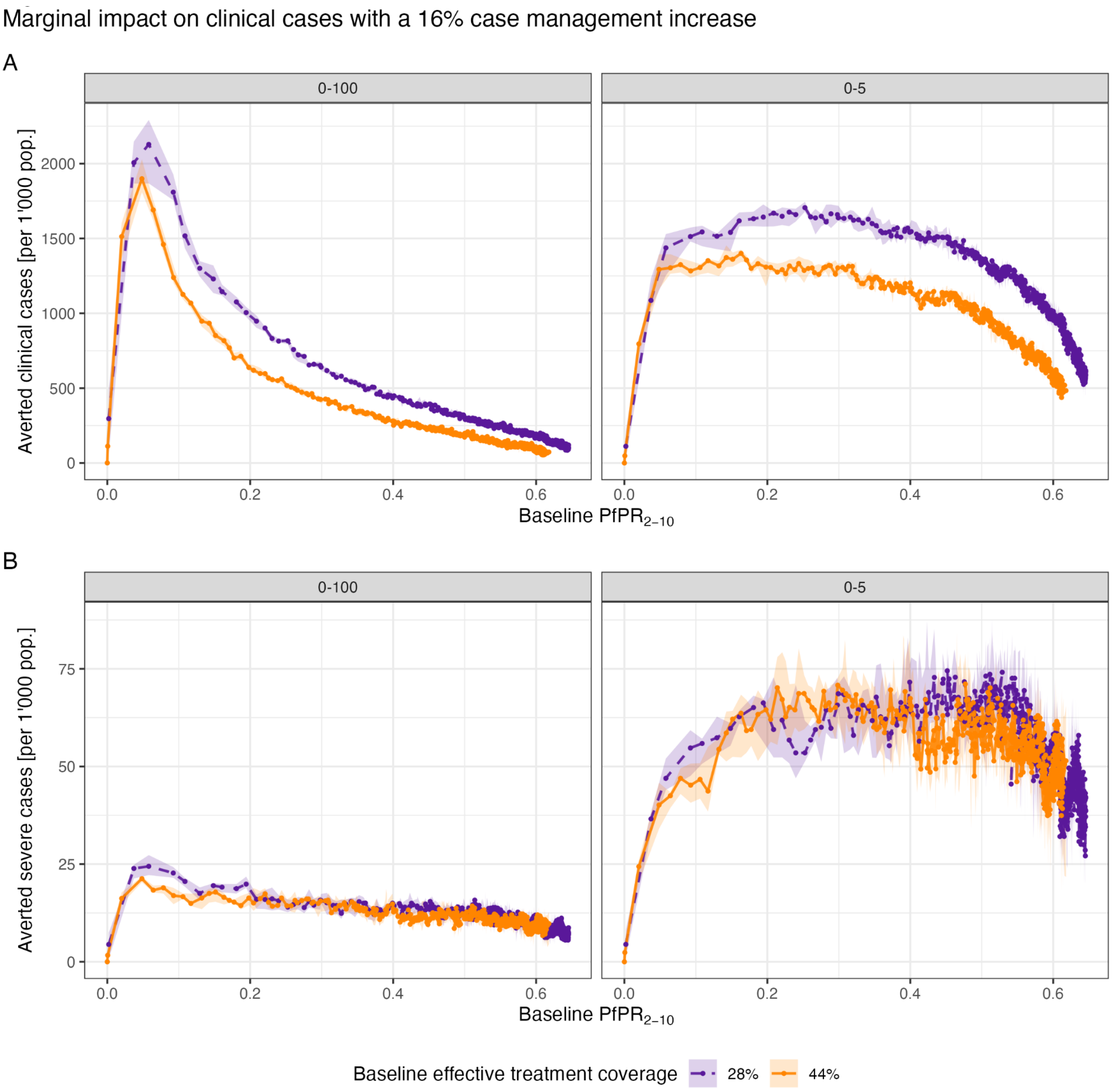

Figure S8. Relationship between cases averted and transmission intensity is nonlinear, and the marginal impact of case management increase is greater at low historical case management levels. Cumulative cases averted per 1'000 population between 2023 and 2031 in total population (left panels) and in children under 5 (right panels). All clinical cases averted (top panels) and severe clinical cases averted (bottom panels) are shown when increasing effective treatment coverage to 44% starting from baseline effective treatment coverage of 28% (purple), and increasing effective treatment coverage to 60% starting from baseline effective treatment coverage of 44% (orange), and the x-axis represent baseline $PfPR_{2-10}$ *(i.e. when case management remains unchanged). Mean and interquartile range are shown for 10 seeds.*

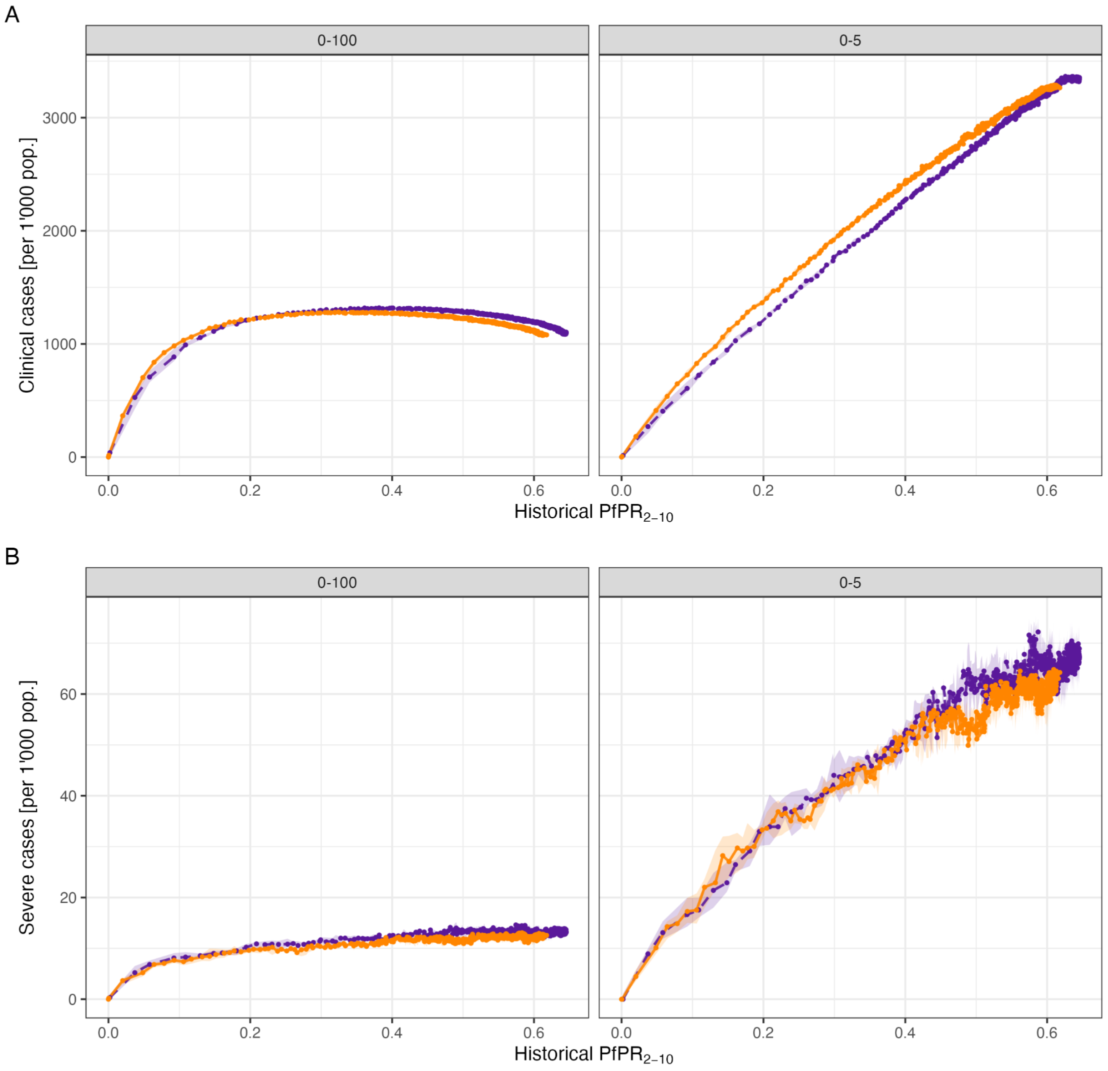

Figure S9. Relationship between cases and transmission intensity is nonlinear. Cases averted per 1'000 population in 2031 in total population (left panels) and in children under 5 (right panels). A) All clinical cases and B) severe clinical cases (bottom panels) are shown when effective treatment coverage are 28% (purple), and 44% (orange), and the x-axis represent baseline $PfPR_{2\text{-}10}$. Mean and interquartile range are shown for 10 seeds.

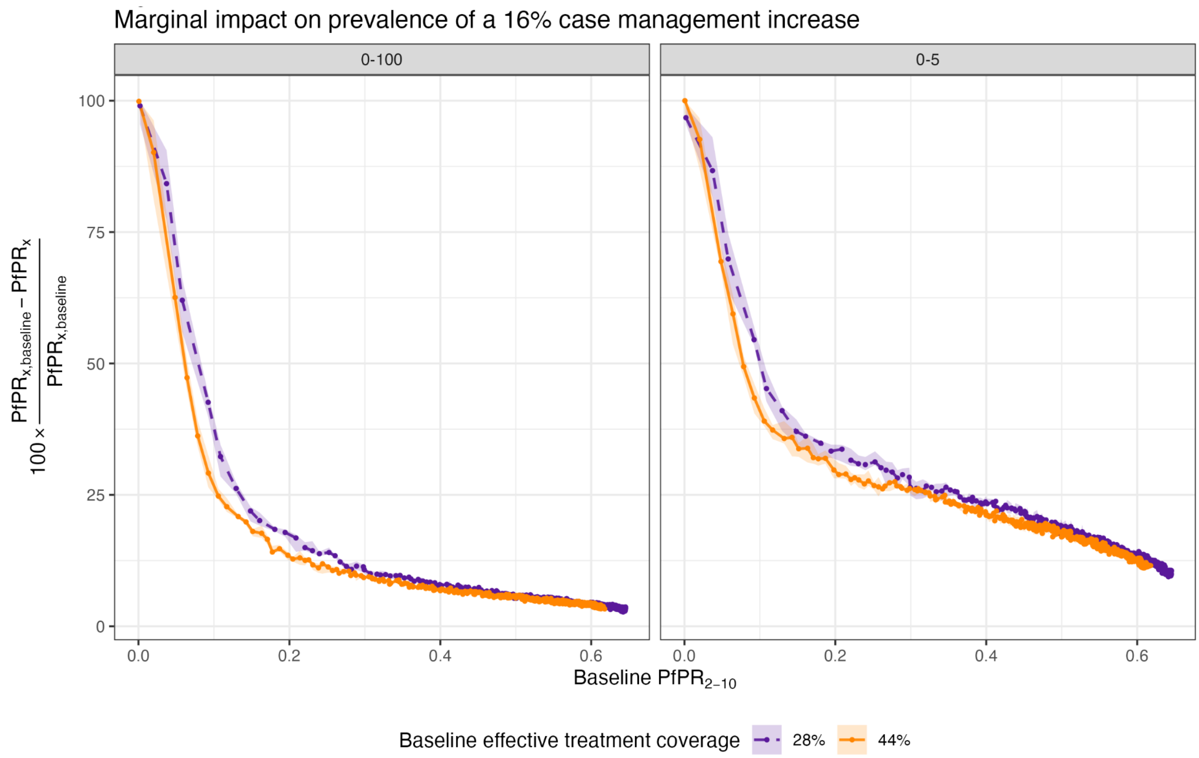

Figure S10. Relationship between prevalence reduction and transmission intensity is nonlinear, and the marginal impact of case management increase is greater at low historical case management levels. Relative prevalence reduction, with $PfPR_x$, baseline prevalence with no change in case management , $PfPR_x$ the prevalence after case management increase, and subscript x the age group (total population or 0-5 years old). Prevalence reduction estimates are for the year 2031, 5 years after case management increased, in function of baseline $PfPR_{2-10}$ (i.e. when case management remains unchanged). The orange line shows the relative reduction when effective treatment coverage increased from 44% to 60%, and the purple line when effective treatment coverage increased from 28% to 44% (both increasing by 16%).

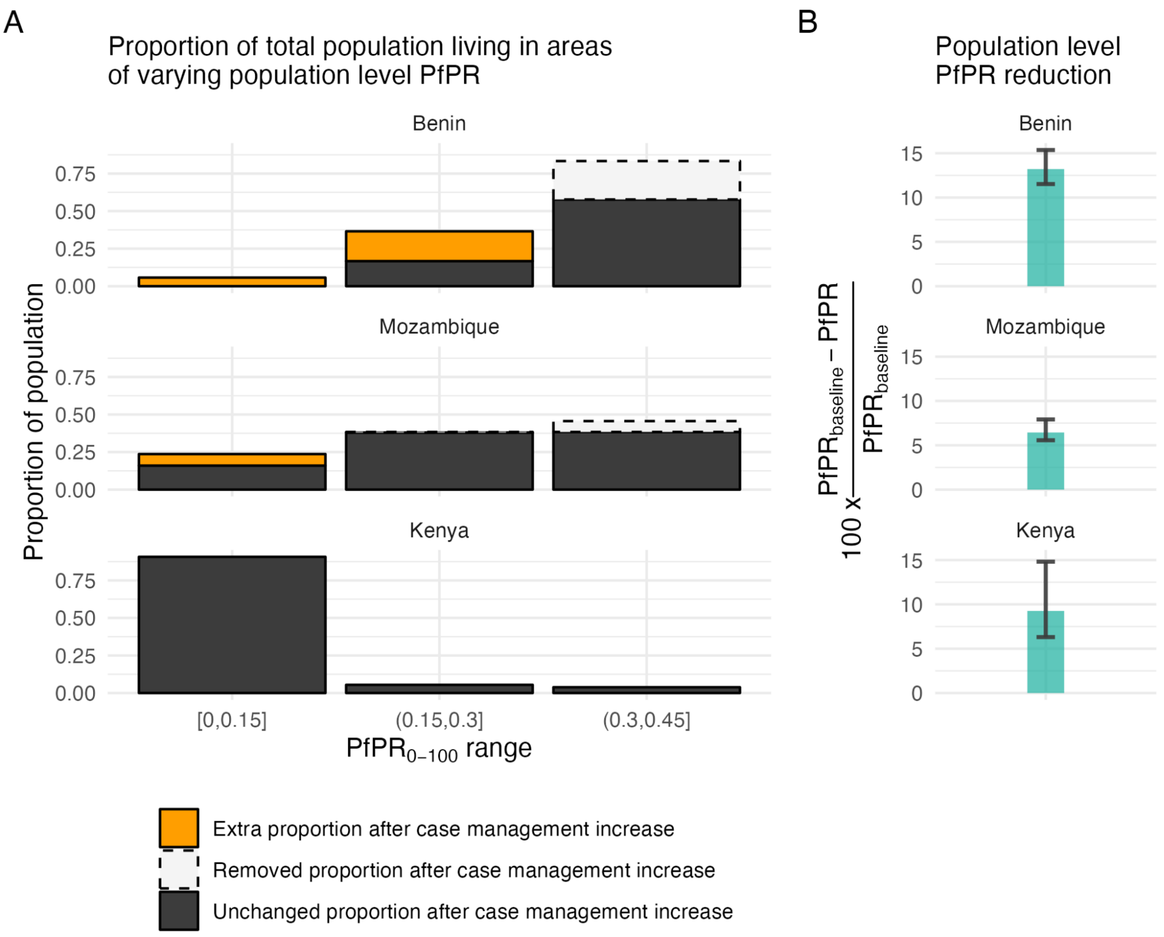

Figure S11. Increasing case management reduces the proportion of the population living in high risk areas. A) Proportion of total population living in regions with low malaria risk (PfPR$_{0\text{-}100}$ less than 0.15), intermediate risk (PfPR$_{0\text{-}100}$ between 0.15 and 0.3), and high risk for malaria (PfPR$_{0\text{-}100}$ greater than 0.3). Solid bars indicate the total proportion of the population living in each area (x-axis) in 2031 – with the orange bars the proportion of the population who were not in given area prior case management increase – and dashed bars indicate the proportion of population that was living in given area prior case management increase but do no longer live in given area after case management increase. Mean across 10 seeds is shown. B) Relative reduction in prevalence in total population (PfPR$_{0\text{-}100}$), with PfPR$_{baseline}$ prevalence with no change in case management and PfPR the prevalence after case management increase. Prevalence estimates are for the year 2031, 5 years after case management increase. Mean, minimum, and maximum across 10 seeds is shown. A-B) Top to bottom panels show results for Benin, Mozambique, and Kenya.

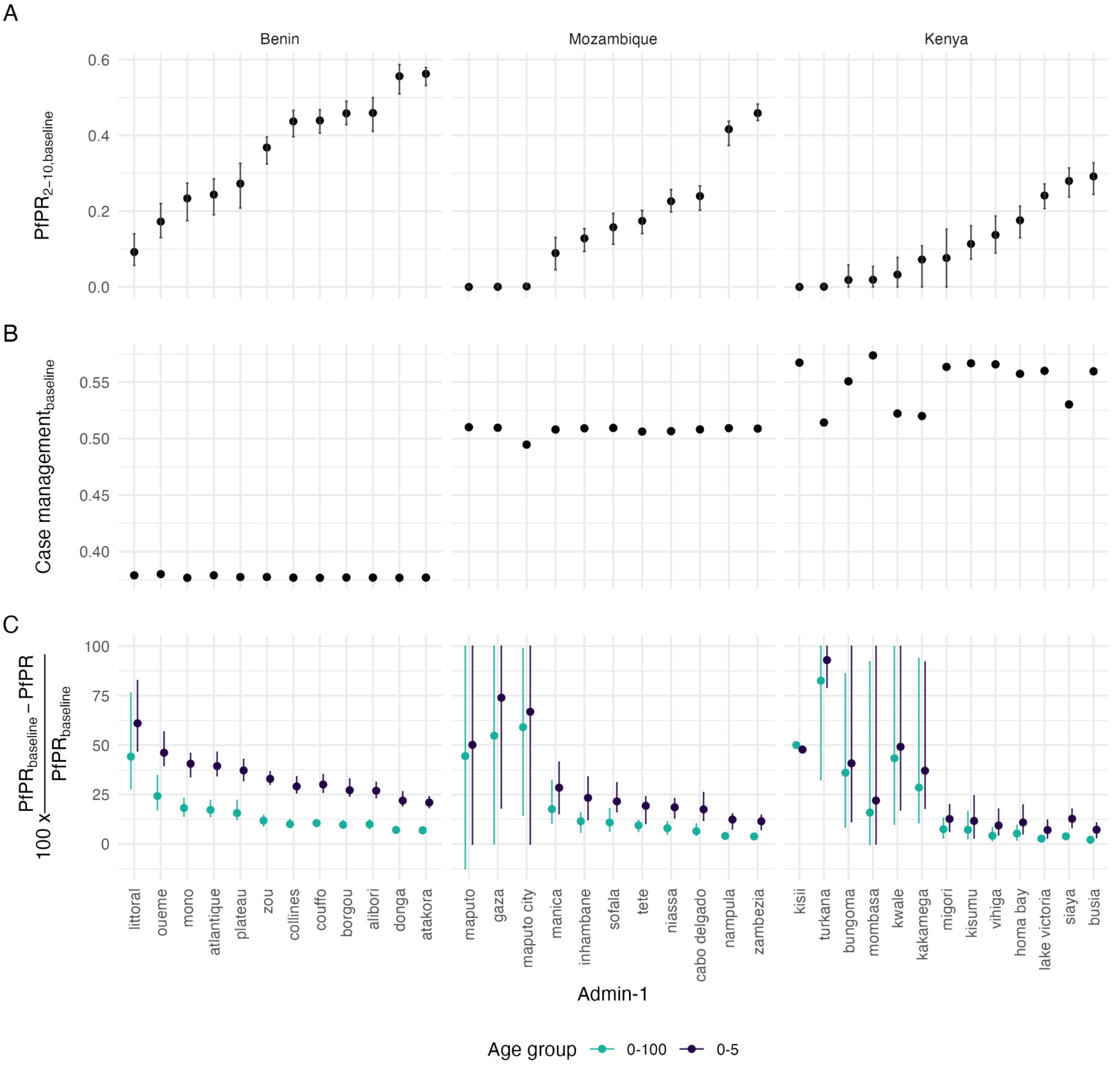

Figure S12. Relative prevalence reduction in Benin, Mozambique, and Kenya at sub-national level. A) Baseline $PfPR_{2\text{-}10}$ (i.e. when case management is not improved) for all admin-1 regions where non zero $PfPR_{2\text{-}10}$ was observed in the simulations, B) Effective access to care estimates (from MAP), and C) Relative reduction in prevalence in 2031, 5 years after case management increased. Reduction is shown for prevalence in children under 5 years old (dark purple) and across the entire population (green). In A) and C), mean, min, and max, across 10 iteration of three calibrated EIR levels (total 30 simulations) are shown.

Supplementary tables

			Relative prevalence reduction (%)	
EIR Category	Age Group	Baseline effective treatment coverage [%]	Mean [min;max]	Median [q.25;q.75]

< 8	under 5 y.o.	28	69 [50.2 ; 100]	62.27 [55.85 ; 83.16]
		44	50.82 [-50.07 ; 100]	39.3 [34.02 ; 60.89]
		54	30.59 [-8.7 ; 100]	15.94 [10.41 ; 32.86]
	Population level PfPR	28	52 [23.38 ; 100]	40.24 [30.55 ; 74.62]
		44	40.99 [13.12 ; 100]	26.31 [18.79 ; 53.98]
		54	30.72 [0.71 ; 100]	11.46 [7.17 ; 39.65]
[8 - 128]	PfPR under 5 y.o.	28	43.28 [35.02 ; 56.1]	42.47 [39.91 ; 46.63]
		44	25.19 [16.68 ; 34.88]	24.86 [22.9 ; 27.46]
		54	9.96 [1.23 ; 18.74]	9.93 [7.78 ; 12.13]
	Population level PfPR	28	17.26 [11.94 ; 28.56]	16.6 [14.73 ; 19.4]
		44	9.49 [5.3 ; 18.49]	9.14 [7.8 ; 10.84]
		54	3.66 [-0.39 ; 9.43]	3.55 [2.43 ; 4.74]
[32 - 320]	PfPR under 5 y.o.	28	25.95 [15.03 ; 42.68]	25.06 [22.02 ; 29.34]
		44	14.67 [5.27 ; 27.56]	14.32 [12.11 ; 17.08]
		54	5.89 [-2.75 ; 16.34]	5.87 [3.9 ; 8.04]
	Population level PfPR	28	8.78 [3.88 ; 16.64]	8.51 [7.52 ; 9.86]
		44	4.71 [0.86 ; 10.19]	4.68 [3.85 ; 5.53]
		54	1.79 [-1.37 ; 5.46]	1.78 [1.09 ; 2.52]

Table S1. Relative prevalence reduction in archetype simulations.

			Relative incidence reduction (%)	
EIR Category	Age Group	Baseline effective treatment coverage [%]	Mean [min;max]	Median [q.25;q.75]
< 8	Under 5 y.o.	28	50.83 [22.61 ; 100]	40.2 [30.23 ; 70.82]

		44	27.03 [-1500.71 ; 100]	22.25 [16.02 ; 51.52]
		54	25.06 [-12.88 ; 100]	7 [2.94 ; 23.01]
	Population level	28	40.58 [10.18 ; 100]	25.74 [17 ; 64.67]
		44	30.6 [-32.14 ; 100]	12.45 [6.84 ; 41.59]
		54	22.76 [-12.5 ; 100]	1.64 [-1.31 ; 26.2]
[8 - 128]	Under 5 y.o.	28	17.61 [10.27 ; 28.3]	16.95 [14.97 ; 19.79]
		44	8.41 [2.1 ; 18.05]	8.15 [6.62 ; 9.86]
		54	2.78 [-3.45 ; 9.54]	2.56 [1.64 ; 3.97]
	Population level	28	7.47 [2.77 ; 15.66]	7.17 [5.62 ; 8.78]
		44	2.54 [-1 ; 7.37]	2.57 [1.6 ; 3.31]
		54	-0.36 [-3.28 ; 3.34]	-0.33 [-1.08 ; 0.3]
[32 - 320]	Under 5 y.o.	28	6.54 [-0.01 ; 16.45]	6.08 [4.66 ; 8.02]
		44	3.16 [-2.25 ; 10]	3.07 [1.97 ; 4.17]
		54	1.21 [-3.04 ; 6.31]	1.17 [0.3 ; 2.08]
	Population level	28	2.1 [-2.38 ; 7.59]	2.05 [1.21 ; 2.99]
		44	0.66 [-3.98 ; 4.3]	0.69 [-0.09 ; 1.4]
		54	-0.01 [-4.01 ; 3.53]	-0.01 [-0.73 ; 0.76]

Table S2. Relative incidence reduction in archetype simulations.

			Cases Averted per 1'000 population			
			All cases		Severe Cases	
EIR Category	Age Group	Tmt cov.* [%]	Mean [min;max]	Median [q.25;q.75]	Mean [min;max]	Median [q.25;q.75]
< 8	Under 5 y.o.	28	2411.6 [137.37 ; 3275.32]	2683.11 [2367.52 ; 2872.13]	90.73 [2.38 ; 170.02]	98.7 [71.81 ; 113.13]
		44	1010.36 [-14.27 ; 1679.38]	1151.68 [884.14 ; 1290.91]	40.43 [-7.71 ; 88.65]	43.41 [20.96 ; 60.02]

		54	312.12 [-122.8 ; 1037.97]	301.82 [138.63 ; 461.48]	13.28 [-38.65 ; 66.54]	10.12 [0.45 ; 25.6]
	Pop. level	28	2030.92 [373.44 ; 3573.11]	1895.22 [1549.94 ; 2498.5]	31.04 [3.33 ; 46.89]	32.89 [27.69 ; 36.94]
		44	862.55 [-17.22 ; 2015.22]	784.22 [600.61 ; 1135.36]	13.93 [0 ; 27.33]	15.33 [10.28 ; 18.36]
		54	216.86 [-202 ; 1608.56]	110.44 [11.03 ; 326.11]	4.23 [-6.44 ; 17]	3.67 [0.44 ; 6.92]
[8 - 128]	Under 5 y.o.	28	2776.54 [2268.49 ; 3254.7]	2778.4 [2654.31 ; 2900.88]	124.6 [57.63 ; 218.76]	123.1 [104.67 ; 141.41]
		44	1193.42 [646.32 ; 1672.08]	1188.71 [1072.32 ; 1295.79]	64.22 [12.38 ; 139.86]	63.07 [45.98 ; 79.64]
		54	367.21 [-238.2 ; 816.93]	375.42 [259.38 ; 480.81]	24.53 [-46.38 ; 110.59]	23.07 [8.5 ; 38.13]
	Pop. level	28	866.44 [516.22 ; 1479]	822.28 [708 ; 993.5]	27.7 [13.33 ; 46.56]	27.67 [23.03 ; 31.56]
		44	324.88 [70.33 ; 693.11]	317.11 [255.89 ; 386.69]	14.27 [0.33 ; 31.22]	14.67 [10.44 ; 17.67]
		54	13.99 [-221.33 ; 219.78]	17.83 [-27.86 ; 58.75]	5.55 [-6.89 ; 22.89]	5.5 [2.33 ; 8.64]
[32 - 320]	Under 5 y.o.	28	1668.53 [527.01 ; 3076.57]	1605.19 [1318.63 ; 2012.21]	104.71 [21.36 ; 193.87]	103.48 [81.41 ; 127.25]
		44	763.44 [-71.16 ; 1578.07]	758.86 [574.21 ; 952.41]	51.54 [-24.43 ; 137.78]	51.62 [33.28 ; 69.18]
		54	270.25 [-364.62 ; 961.59]	273.1 [138.15 ; 403.75]	17.72 [-58.22 ; 101.64]	18.33 [-3.6 ; 38.62]
	Pop. level	28	297.55 [-32.67 ; 766]	273.67 [207.53 ; 367.58]	20.03 [3.89 ; 40.11]	19.78 [15.56 ; 24.33]
		44	113.23 [-103.56 ; 403.22]	111.33 [67.44 ; 158.36]	9.86 [-5.67 ; 26.33]	9.89 [6.11 ; 13.33]
		54	22.14 [-163.11 ; 211.44]	21.89 [-15.11 ; 59.47]	3.58 [-13.11 ; 21.78]	3.56 [-0.56 ; 7.67]

*Table S3. Cases averted in archetype simulations. Cumulative cases averted per 1'000 population between 2023 and 2031 when increasing access to effective treatment coverage from baseline coverage to 60%. * Tmt cov. : Baseline effective treatment coverage.*

	Population level			Under 5 y.o.			2-10 y.o.		
	PfPR [%]		% reduction	PfPR [%]		% reduction	PfPR [%]		% reduction
	BAU	CM increase		BAU	CM increase		BAU	CM increase	
Benin	35.2<br>32.3;37.5]	30.6<br>[27.4;33.2]	12.92<br>[15.07;11.51]	26.1<br>[22.5;29]	18.5<br>[15.5;20.9]	29.11<br>[31.31;27.84]	35<br>[30.8;38.6]	26.2<br>[22.3;29.4]	24.99<br>[27.42;23.90]

Kenya	4.2 [2.9;5.7]	3.8 [2.6;5.2]	9.45 [11.04;8.74]	1.8 [1.2;2.6]	1.6 [1;2.2]	13.74 [14.9;13.35]	2.6 [1.7;3.6]	2.2 [1.5;3.1]	13.46 [16.05;13.5]
Mozambique	26.9 [24.4;29]	25.2 [22.6;27.3]	6.29 [5.83; 7.35]	17.2 [15;19.1]	14.8 [12.6;16.5]	13.97 [13.62;15.69]	24.4 [21.5;26.8]	21.5 [18.8;23.8]	11.84 [11.04;12.24]

Table S4. Prevalence and relative prevalence reduction in country specific simulations. Relative prevalence reduction computed as 100 $*(PfPR_{baseline}-PfPR)/PfPR_{baseline}$, with $PfPR_{baseline}$ prevalence with no change in case management and PfPR the prevalence after case management increase

	Population level			Under 5 y.o.			2-10 y.o.		
	Incidence [per 1000]		% reduction	Incidence [per 1000]		% reduction	Incidence [per 1000]		% reduction
	BAU	CM increase		BAU	CM increase		BAU	CM increase	
Benin	1174 [1145;1196]	1085 [1032;1123]	75.834 [98.754;61.23]	2183 [1998;2319]	1926 [1744;2079]	117.413 [127.331;103.672]	2162 [2041;2258]	1971 [1806;2093]	88.38 [115.161;72.904]
Kenya	190 [108;249]	175 [103;235]	79.051 [44.64;57.269]	205 [124;275]	188 [118;253]	81.051 [53.578;77.068]	243 [146;322]	224 [139;302]	79.166 [46.281;61.77]
Mozambique	985 [944;1026]	952 [910;990]	33.273 [35.597;35.218]	1647 [1511;1741]	1562 [1433;1664]	51.394 [51.635;44.286]	1730 [1613;1825]	1663 [1553;1746]	38.63 [37.541;43.273]

Table S5. Incidence and relative incidence reduction in country specific simulations

	Population level			Under 5 y.o.			2-10 y.o.		
	Averted per 1’000 pop.		Population	Averted per 1’000 pop.		Population	Averted per 1’000 pop.		Population
	Cases	Severe cases		Cases	Severe cases		Cases	Severe cases	
Benin	619.88 [540.74;728.86]	15.41 [13.83;18.21]	15672310	1682.33 [1628.35;1742.6]	74.25 [65;83.33]	2580127.65	1372.03 [1254.51;1526.42]	30.67 [24.14;39.6]	3458744.21
Kenya	81.39 [25.93;108.81]	1.03 [0.45;1.34]	66449655	91.49 [39.61;125.16]	3.06 [1.74;4.1]	9819111.37	106.59 [37.8;147.66]	2.04 [0.89;2.73]	13773540
Moz.	237.48 [205.31;287.12]	5.98 [4.91;7.15]	41184834	555.58 [494.5;588.67]	26.3 [20.81;32.63]	7119548.24	484.81 [427.4;542.99]	12.65 [9.47;18.73]	9318740.76

Table S6. Cases averted in country specific simulations. Cumulative cases averted per 1’000 population between 2023 and 2031 when increasing access to treatment from historical access to 60% coverage.
